# Fine-Tuning Donor Material Deposition with Ultrasonic Aerosol Jet Printing to Balance Efficiency and Stability in Inverted Organic Photovoltaic Devices

**DOI:** 10.1021/acsami.5c09318

**Published:** 2025-07-31

**Authors:** Vanessa Arango-Marín, Jonas Wortmann, Tobias Osterrieder, Paul Weitz, Juan S. Rocha-Ortiz, Mingjian Wu, Xin Zhou, Fabian Eller, Thomas Heumüller, Jens A. Hauch, Chao Liu, Vincent M. Le Corre, Erdmann Spiecker, Eva M. Herzig, Guanghao Lu, Larry Lüer, Christoph J. Brabec

**Affiliations:** † Department of High Throughput Methods in Photovoltaics, 28334 Forschungszentrum Jülich GmbH, Helmholtz-Institute Erlangen-Nürnberg (HI ERN), Immerwahrstraße 2, Erlangen 91058, Germany; ‡ Department of Materials Science and Engineering, Institute of Materials for Electronics and Energy Technology (i-MEET), Friedrich-Alexander-Universität Erlangen-Nürnberg, Martensstraße 7, Erlangen 91058, Germany; § Centre for Advanced Photovoltaics and Thin-film Energy Devices (CAPE), 6174 University of Southern Denmark, Alsion 2, Sønderborg DK-6400, Denmark; ∥ Institute of Micro- and Nanostructure Research(IMN) & Center for Nanoanalysis and Electron Microscopy (CENEM), Interdisciplinary Center for Nanostructured Films (IZNF), Cauerstraße 3, Erlangen 91058, Germany; ⊥ Dynamics and Structure Formation − Herzig Group, University of Bayreuth, Universitätsstraße 30, Bayreuth 95447, Germany; # Institute of Science and Technology, Xi’an Jiaotong University, Xi’an 710054, China

**Keywords:** aerosol jet printing, organic solar cells, ultrasonic spray coating, sequential deposition, polymer deposition

## Abstract

The response surface methodology (RSM) based on a Box–Behnken (BB) design of experiment (DoE) approach was performed, with the central point repeated four times to enhance statistical reliability, to systematically investigate the influence of ultrasonic aerosol jet printing (uAJP) parameters such as speed, flow, and power, while depositing the donor material deposition, on the acceptor/donor ratio and power conversion efficiency (PCE). Efforts were made to tune the D:A ratio to approximately 1:1.2, a composition widely used for the PM6:Y12 active layer system. Despite the sequential deposition of the donor material onto the acceptor, the resulting active layer exhibited a bulk heterojunction (BHJ) morphology rather than a layer-by-layer (LbL) structure. Further analysis such as film-depth-dependent light absorption spectra (FLAS) and cross section of the electron energy-loss spectroscopy (EELS) in a scanning transmission electron microscope (STEM) or STEM-EELS was used to explore the interplay between deposition parameters and vertical blending behavior in the active layer. Finally, we evaluated the stability of these OPV devices under continuous one-sun illumination for 1080 h, revealing that the most efficient devices also exhibited the highest operational stability.

## Graphical abstract

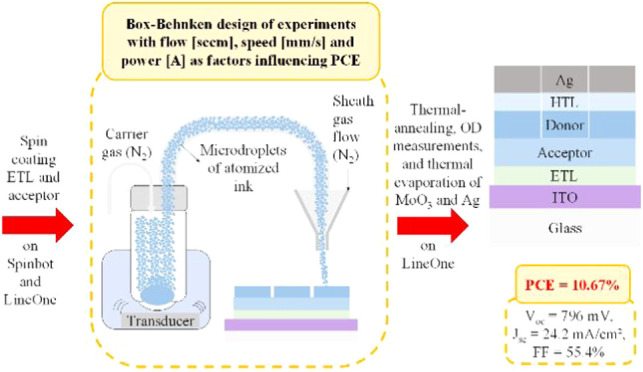

## Introduction

Achieving net-zero carbon emissions by 2050 requires sustainable energy solutions, with photovoltaic (PV) technology playing a crucial role in meeting the increasing global energy demand.^1^ Few studies in PV have employed the Box–Behnken design (BBD) for process optimization, including Ag nanoparticle ink formulation for aerosol jet printing (AJP)^2^ and improving the power conversion efficiencies (PCEs) in dye-sensitized solar cells.^3^ Among PV technologies, organic photovoltaics (OPVs) stand out due to their potential for significant cost reduction,^4^ as well as their eco-friendly manufacturing process, cost-effectiveness, scalability, flexibility, and semitransparent properties.^5,6^ Nowadays, the PCEs of OPV devices have reached values beyond 19%, particularly with the development of small-molecule nonfullerene acceptors (NFAs). However, achieving the optimal morphology remains a key challenge in ensuring both efficiency and stability in OPV device production.^7^

A promising strategy to overcome this challenge involves sequential deposition or layer-by-layer (LbL) deposition of NFAs onto the donor material in a conventional OPV structure, a method that has demonstrated high PCEs.^8,9^ For instance, Sun et al. found that LbL facilitated the formation of larger, well-separated donor–acceptor domains, resulting in a more stable morphology compared to bulk heterojunction (BHJ) blends using blade coating.^10^ Later, Zhan et al. reported efficiencies exceeding 18% for OPV devices by integrating LbL with a ternary strategy using spin coating.^11^

In normal-structure OPV devices, layer-by-layer vertical blending is easier to achieve than in inverted OPVs. In the normal structure, the polymer donor is deposited first and forms a gel-like network upon solvent evaporation, preventing the dissolution of the subsequently deposited NFA. Additionally, the rigid conjugated cores and strong π–π interactions of NFAs reduce their miscibility with the underlying polymer. In contrast, in inverted OPVs, the donor is deposited after the acceptor and its flexible backbone makes it more prone to dissolving into the loosely packed NFA layer. This challenge complicates vertical stratification in inverted n–i–p structures, as the donor material can easily mix with or dissolve into the underlying NFA. While normal-structure OPVs typically achieve higher PCE values, they generally exhibit lower stability compared to that of their inverted counterparts. Given the advantages of inverted structures, investigating sequentially deposited inverted OPV devices is of great interest. Recently, Wang et al. successfully employed transfer-printing technique to achieve bilayer vertical stacking in inverted OPV devices using PM6:IT-4F as the active layer.^12^

Among the various deposition methods explored for OPV fabrication, aerosol jet printing (AJP) stands out for its unique combination of versatility, scalability, and precision. In contrast to spin coating, which is not a scalable technique due to substrate size limitations and significant waste of expensive materials, or blade coating, which despite being compatible with large-area fabrication, typically requires direct substrate contact and often involves postprocessing steps, AJP offers noncontact, high-resolution patterning and fine control over film thickness and morphology via tunable adjustable deposition parameters. It allows a broad range of ink viscosities, supports deposition on flat and complex substrates, and is compatible with roll-to-roll manufacturing. These attributes make AJP a promising technique for the scalable, reproducible, and environmentally friendly manufacturing of next-generation OPV devices.^13,14^

Although several studies have demonstrated that sequential deposition can effectively control the active layer morphology in normal-structure OPV devices,^8,9,15^ yielding active layers with 60–80% of their thickness presenting BHJ structure while the rest of the thickness was mostly close to either pristine positive or negative regions; its implementation on inverted-structure OPVs with the PM6:Y12 active layer system remains scarcely explored, particularly in the context of aerosol jet printing as deposition technique. To the best of our knowledge, no studies have explored BBD to optimize the sequential deposition of the donor via aerosol jet printing on inverted OPV devices. Therefore, in this work, we employ a BBD approach to investigate the effects of ultrasonic aerosol jet printing parameters such as speed, flow, and power while depositing the donor material; on the donor/acceptor ratio and power conversion efficiency (PCE) as response variables on inverted OPV devices. Additionally, we analyzed the vertical phase distribution in the active layer under certain device conditions and evaluated the stability of the inverted OPV devices.

## Methods

### Materials

We used the same indium tin oxide (ITO) substrates and the ZnO (N10) as electron transport layer (ETL) treated as explained in a previous study of our group.^16^ The ITO was purchased from Liaoning Yike Precision New Energy Technology Co., Ltd. and the ZnO from Avantama AG. All materials from the active layer were used as received; the poly­[(2,6-(4,8-bis­(5-(2-ethylhexyl-3-fluoro)­thiophen-2-yl)-benzo­[1,2-b:4,5-b’]­dithiophene))-*alt*-(5,5-(1′,3′-di-2-thienyl-5′,7′-bis­(2-ethylhexyl)­benzo­[1′,2′-c:4′,5′-c′]­dithiophene-4,8-dione))] or PM6 was purchased from Solamer Materials, the 2,2′-((2Z,2′Z)-((12,13-bis­(2-butyloctyl)-3,9-diundecyl-12,13-dihydro-[1,2,5]­thiadiazolo­[3,4-*e*]­thieno­[2″,3″:4′,5′]­thieno­[2′,3′:4,5]­pyrrolo­[3,2-*g*]­thieno­[2′,3′:4,5]­thieno­[3,2-*b*]­indole-2,10-diyl)­bis­(methanylylidene))­bis­(5,6-difluoro-3-oxo-2,3-dihydro-1H-indene-2,1-diylidene))­dimalononitrile or Y12 from 1-material, the o-xylene, the MoO3, and the Ag from Sigma-Aldrich.

### Equipment

While the Spinbot^17^ and LineOne^18^ setups are described in other publications from our research group, the ultrasonic aerosol jet printing (uAJP) process was performed using the AJ300 printer from Optomec. We used the LineOne to obtain the *J–V* parameters in the dark and under AM1.5 G, employing the SINUS-70 solar simulator from Wavelabs and to obtain the optical density (OD) as reported elsewhere.^19^ The film thicknesses were measured by using the profilometer P7 from Tencor. The cross-sectional lamella of the OPV device was prepared within a dual-beam FIB-SEM Helios NanoLab 660 (Thermo Fischer Scientific, TFS) following the standard lift-out routine. Scanning transmission electron microscopy (STEM) investigation on the lamella was performed using a TFS double Cs-corrected Titan Themis microscope operated at 300 kV. High spatial resolution electron energy-loss spectroscopy (EELS) was acquired using the GIF Quantum ERS. Furthermore, to obtain the cross-sectional STEM-EELS images, we used the following conditions: a convergence half-angle of 15.7 mrad, a camera length of 29 mm, a probe current between 80 and 150 pA, a sampling size (i.e., pixel size) of 0.8–1.5 nm/pixel, and a dwell time between 2 and 4 ms.^17^ Moreover, the spectrometer was set to DualEELS mode, and the dispersion of the EELS spectrometer was set to 0.5 eV/channel. Under these conditions, good-quality EELS spectra suitable for S–K and C–K analyses are obtained, while the electron beam-induced damage is evaluated to be negligible.^12^ The conditions under which the films were performed to measure FLAS were 20 mm/s, 0.475 A, and 100 sccm and to measure the cross-sectional STEM-EELS were 10 mm/s, 0.475 A, and 100 sccm. Moreover, grazing incidence wide-angle X-ray scattering (GIWAXS) was performed on a laboratory system at the University of Bayreuth (Xeuss 3.0, Xenocs SAS, Grenoble, France) with a Cu Kα source (λ = 1.54 Å), a Dectris EIGER 2R 1 M detector, a sample-to-detector distance of 72 mm, and a beam size of 500 μm. Scattering experiments were carried out at room temperature under a vacuum on the thin film between the electrodes of full devices. The sample length in the beam direction was 5 mm. The incident angle was set to 0.18° (above the critical angle of ∼0.16°), which probes the full depth of the films. The presented q-profiles are cake cuts covering an azimuthal angle of 70–110° for the cuts in the vertical direction and 0–20°, as well as 160–180° for the cuts in the horizontal direction.

### Device Fabrication

The ITO/glass substrates were sequentially cleaned via sonication for 10 min each in deionized water (DIW), acetone, and isopropanol (IPA). The Y12 (acceptor material) and PM6 (donor material) solutions were mechanically stirred at 600 rpm overnight at 80 °C under a nitrogen atmosphere, with concentrations of 25 and 7.5 mg/mL in o-xylene, respectively. We selected o-xylene as a solvent for the solutions due to its classification as a green solvent and proven compatibility with the PM6:Y12 system, enabling high device efficiencies. The Y12 solution concentration of 25 mg/mL was chosen based on standard formulations commonly used in high-performance OPV devices with this material system.^19^ In contrast, the PM6 concentration of 7.5 mg/mL was determined through a rapid trial-and-error screening. Lower concentrations led to excessive removal of the underlying acceptor layer during deposition, while higher concentrations demanded substantially more power for aerosolization during the AJP.

All OPV device fabrication steps performed in the LineOne setup were conducted under a nitrogen atmosphere, while those carried out outside of it were performed in air. On the cleaned ITO substrates, we spin-coated the ZnO using 60 μL of solution at 2500 rpm, followed by annealing at 200 °C for 30 min using the Spinbot system. The samples were then transferred to the LineOne setup, where the acceptor layer was deposited by spin coating 30 μL of the Y12 solution at 800 rpm for 40 s. This layer was subsequently annealed at 120 °C for 2 min.

Subsequently, the samples were transferred to the AJ300 printer for deposition of the donor material (PM6 solution) onto the acceptor layer. Following donor deposition, the samples were returned to the LineOne setup for thermal annealing of the active layer at 120 °C for 3 min and subsequent absorption measurements. Afterward, the hole transport layer (HTL), consisting of 10 nm of MoO_3_, and the top electrode, comprising 100 nm of Ag, were deposited via thermal evaporation under a nitrogen atmosphere. The samples were transferred again to LineOne to measure the *J–V* characteristics. An additional reference device was produced via uAJP with PM6:Y12 ink for comparison. Then, the stability of the OPV devices under continuous one-sun illumination and 20 °C for 1080 h was monitored, following a similar method to that described in reference 20. Finally, the devices were transferred to LineOne to measure the *J–V* characteristics after the degradation test.

## Results and Discussion

Aerosol jet printing (AJP) has emerged as a novel and scalable deposition method used in the production of optoelectronic devices,^21–23^ especially of OPV devices.^16,24–30^ The power conversion efficiency (PCE) in these devices is influenced nonlinearly by printing parameters such as power, flow, and speed, often resulting in a complex response surface with a peak efficiency region. To efficiently capture these nonlinear interactions and optimize the process, we employed the Box–Behnken design (BBD), which uses a quadratic model while minimizing the number of experiments and avoiding extreme or impractical conditions. The workflow for fabricating inverted OPV devices using ultrasonic aerosol jet printing (uAJP) to deposit the donor material in the active layer is illustrated in Figure 1. Additionally, the constant parameters used during donor layer deposition are listed in Table 1.

**Figure 1:**
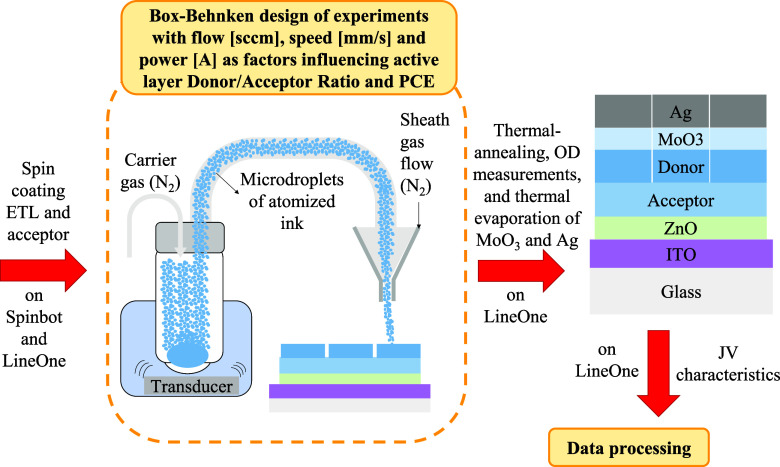
Workflow to fabricate the inverted-structure organic photovoltaic devices with the donor material deposited using ultrasonic aerosol jet printing and Box–Behnken design of experiments.

**Table 1:** Aerosol Jet Printing Conditions for Depositing a PM6 Solution in OPV Devices

**AJP conditions**	**values**
sheath gas flow [sccm]	200
atomized gas flow [sccm]	(80, 100, 110)
atomized gas pressure [psia]	1.6
atomization current [A]	(0.45, 0.475, 0.5)
area filling type	serpentine
printing speed [mm s^‑1^]	(8, 10, 12)

In this study, the uAJP process was employed to deposit the microdroplets containing the donor material onto the precasted acceptor layer, as shown in Figure 1. Therefore, it produces extensive interfacial areas between the incoming droplets and the acceptor surface for interfacial diffusion prior to their coalescing into a donor layer. As a result, predominantly BHJ morphology is obtained, facilitating local mixing of donor and acceptor molecules. Such intermixing at the interface between the microdroplet and the acceptor film is known to promote the formation of a bulk heterojunction morphology, rather than a distinct bilayer morphology. Moreover, the precise control over droplet size and deposition offered by AJP further influences the degree of intermixing and the resulting active layer morphology.^13,14^

Compared to full-factorial or central composite designs (CCDs), which for 3 factors at 3 levels would require 27 points or devices, the BBD only requires 12 edge points and 3–5 center points, totaling between 15 and 17 devices, by focusing on the edges and center points of the design space. BBD is a demonstrated efficient approach to optimizing printing parameters in solar cell fabrication.^3^ Therefore, we employed the BBD to systematically evaluate the influence of three factors at three levels on the response variables during aerosol jet printing. While utilizing aerosol jet printing with an ultrasonic working principle to deposit the donor material PM6 onto the acceptor material Y12 (both material structures are described in the Supporting Information), we maintained certain parameters constant on the aerosol jet printer. These include a buffer solvent initial volume of 20 mL, an initial ink volume of 2 mL, a platen and ink temperature at 20 °C, a 4 mm distance from the end of the tip to the substrate surface, a single printing pass, a printing angle of 90°, and a 3 mm width tip type. Moreover, the selected factors were tested at low, medium, and high values, with detailed conditions for each inverted OPV device provided in Table 2.

**Table 2:** Ultrasonic AJP Conditions for the OPV Devices Explaining the Values of the Factors to Deposit the Donor Material over the ITO/ZnO/Y12 Stack

**device no**.	**flow [sccm]**	**power [A]**	**speed [mm/s]**
**1**	100	0.45	8
**2**	100	0.50	8
**3**	100	0.45	12
**4**	100	0.50	12
**5**	80	0.45	10
**6**	80	0.50	10
**7**	120	0.45	10
**8**	120	0.50	10
**9**	80	0.475	8
**10**	80	0. 475	12
**11**	120	0. 475	8
**12**	120	0. 475	12
**13**	100	0. 475	10
**14**	100	0. 475	10
**15**	100	0. 475	10
**16**	100	0. 475	10

The pictures of the 16 inverted OPV devices with the uAJP donor material on the active layer are shown in Figure S1. Moreover, Figure 2 illustrates the absorption measurements in optical density (OD) for the active layers of all 16 inverted OPV devices performed under the conditions of Table 2, differentiating the low, medium, and high values of the three factors: flow, power, and speed during ultrasonic aerosol jet printing of the donor material. The absorption measurements of the OPV devices were analyzed using a Python script, similar to that explained elsewhere.^31^ This allowed us to determine the mean intensity values of the donor and acceptor peaks across six pixels per device, as reported in Table 3. Then, the ratio (acceptor/donor) was calculated for each device, dividing the OD intensity acceptor peak mean value by the OD intensity donor peak mean value.

**Figure 2:**
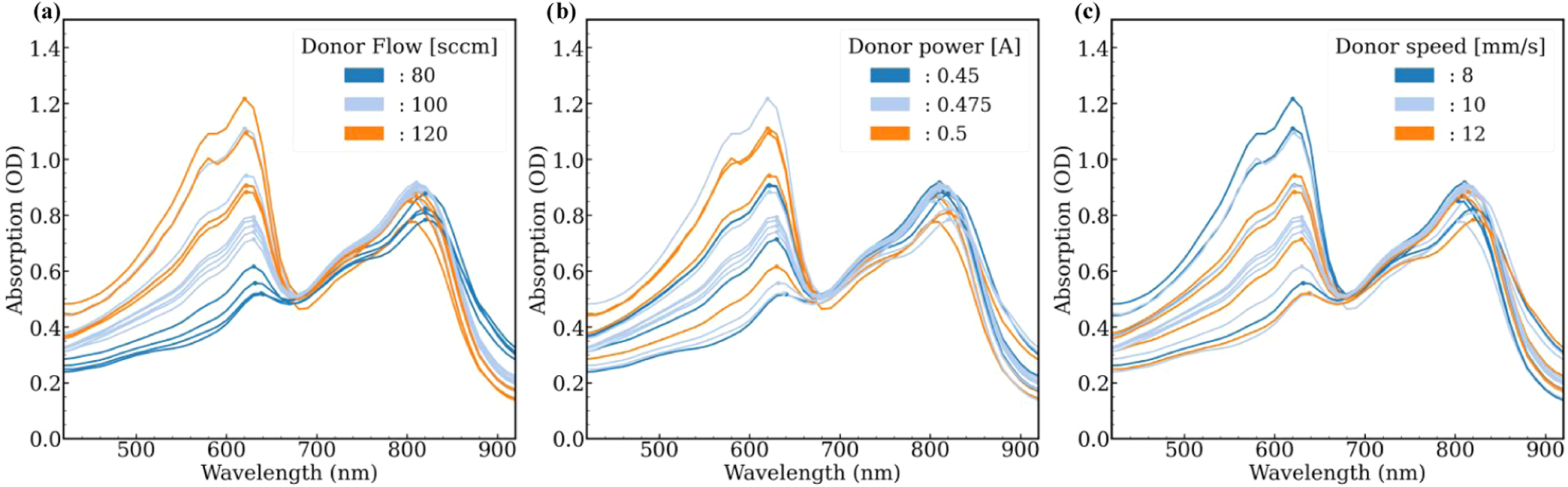
Optical density (OD) or UV–vis absorption measurements of the active layer of the OPV devices after ultrasonic aerosol jet printing of the donor material while screening the (a) flow, (b) power, and (c) speed factors.

**Table 3:** Optical Density (OD) Intensity (Int.) Means of Donor and Acceptor Peaks and Their Ratio per Device

**device no**.	**OD int. donor peak mean**	**OD int. acceptor peak mean**	**ratio (acceptor/donor)**
**1**	0.91	0.92	1.01
**2**	1.11	0.87	0.78
**3**	0.71	0.88	1.24
**4**	0.94	0.89	0.95
**5**	0.52	0.88	1.70
**6**	0.62	0.81	1.31
**7**	0.91	0.89	0.98
**8**	1.10	0.78	0.71
**9**	0.56	0.82	1.48
**10**	0.52	0.78	1.51
**11**	1.22	0.85	0.70
**12**	0.88	0.87	0.98
**13**	0.78	0.91	1.17
**14**	0.76	0.90	1.18
**15**	0.79	0.91	1.14
**16**	0.74	0.89	1.20

### Influence of the Factors on the Acceptor/Donor Ratio

We employed response surface methodology using a Box–Behnken design to examine the effects of flow (F), power (P), and speed (S) in ultrasonic aerosol jet printing on the acceptor/donor ratio and PCE of OPV devices. The coefficients (*x* values) are provided in Figures S11 and S12, respectively.

The predictive models for the response variables, the acceptor/donor ratio, and the PCE follow the form of the eq 1. We developed a Python script for the statistical analysis of the BBD, importing the necessary libraries to implement ordinary least-squares (OLS) regression. The ANOVA results, including model coefficients, *R* ^2^, adjusted *R* ^2^, *p*-values, and *t*-values, are presented in Figures S11 and S12 for the ratio and the PCE, respectively. Additionally, in Figure S2, the effect of the three factors on the donor/acceptor ratio of the OPV devices was studied. Figure S2 shows all of the possible response surface (left side) and contour (right side) plots of (a) flow and power, (b) flow and speed, and (c) power and speed, while holding the third factor at its respective mean value.

1 $$\mathrm{model}=x_{0}+x_{1}*P+x_{2}*S+x_{3}*F+x_{4}*P*S*F+x_{5}P*S+x_{6}*P*F+x_{7}*S*F+x_{8}*\left(P^{2}\right)+x_{9}*\left(S^{2}\right)+x_{10}*\left(F^{2}\right)$$

Shape of the equation for the models to predict ratio and PCE

Figure S2 can be used to predict the factor values required to achieve a desired acceptor/donor ratio in the active layer of OPV devices performed with an ultrasonic aerosol jet printed donor material. For instance, to obtain a D:A ratio of approximately 1:1.2 (acceptor/donor ratio = 1.2), the factor values should fall within the gray-colored region. Ultrasonic aerosol jet printing conditions that produce active layers with higher and lower acceptor concentrations are represented by red and blue regions, respectively. This analysis provides a straightforward model to predict and tune the acceptor/donor ratio in the active layer of an inverted OPV device based on uAJP parameters for the sequential deposition of the donor material on top of the spin-coated acceptor material. Moreover, we printed the donor material under the same BBD conditions over plain glass and then measured the thickness of these films and correlated it to the OD donor peak intensity as shown in Figure S9.

### Influence of the Factors on the PCE

Furthermore, the influence of the factors on the power conversion efficiency (PCE) of the OPV devices is analyzed in Figure 3. The response surface plots (left) and contour plots (right) for the PCE response variable are shown for (a) flow and power, (b) flow and speed, and (c) power and speed, while maintaining the third factor at its mean value and under AM1.5G illumination. Similar figures depicting the response surface plots for the *J*_sc_ are presented in Figure S3. From Figure 3, it can be observed that the highest PCE values for the inverted OPV devices were obtained within the middle levels of all of the factors or center point using 100 sccm for flow, 0.475 A for power, and 10 mm/s for speed.

**Figure 3:**
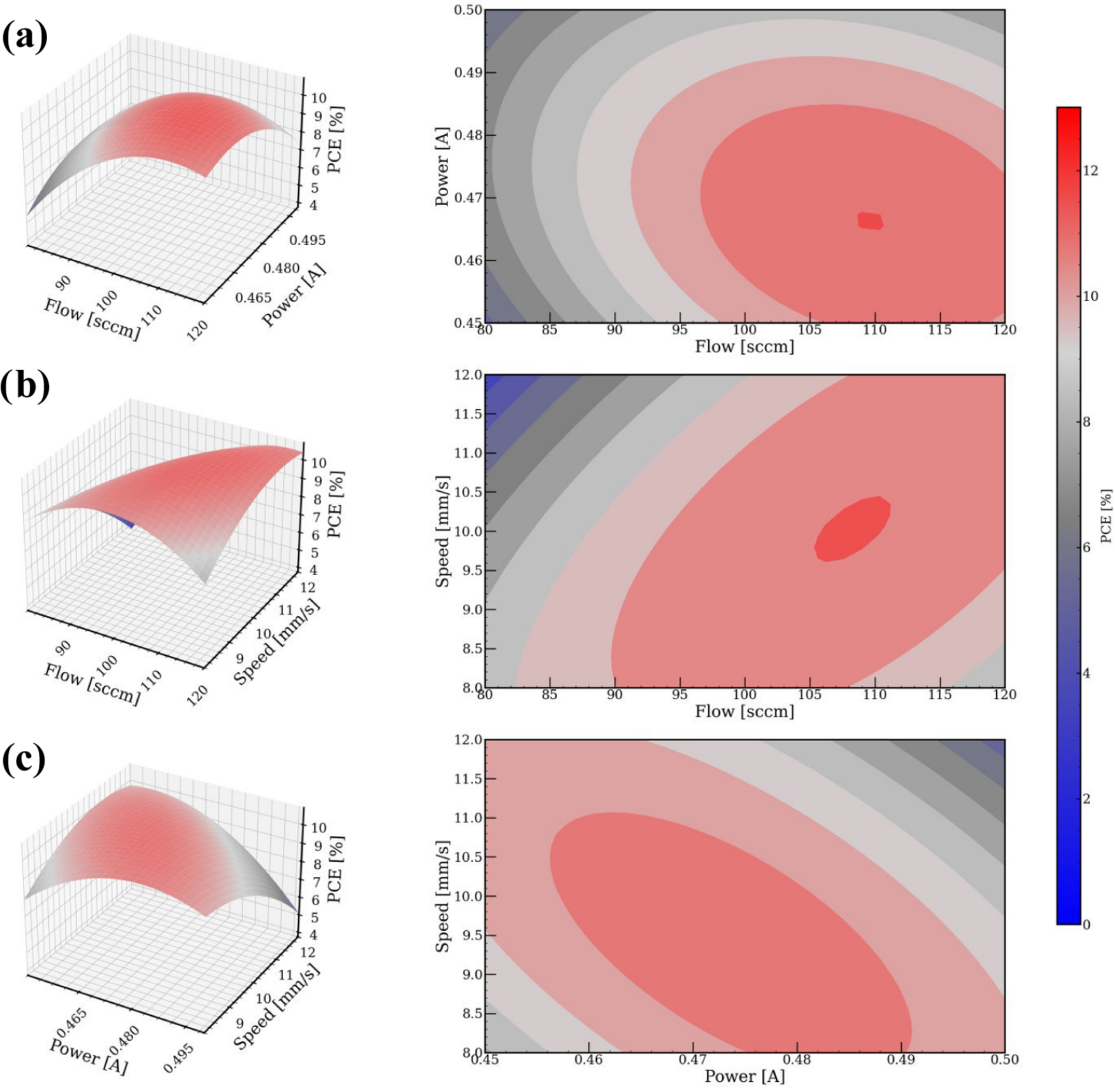
Response surface method with the Box–Behnken design of experiments using PCE as the control variable. Left side: response surface plots and right side: contour plots. Screening two factors of (a) flow and power, (b) flow and speed, and (c) power and speed, while holding the third factor at its respective mean value.

Additionally, the *J–V* curves of the OPV devices based on the factors from left to right flow, power, and speed under (a) AM1.5G and (b) in the dark are presented in Figure S7. The ANOVA results for PCE, including model coefficients, *R* ^2^, adjusted *R* ^2^, *p*-values, and *t*-values, are shown in Figure S12. Similarly, the statistical analysis for *J*_sc_ can be found in Figure S13. The hero device was No. 14 processed under center point conditions; it achieved a PCE of 10.67% ± 0.15, a *V*_oc_ of 796 mV ± 1.3, a *J*_sc_ of 24.2 mA/cm^2^ ± 0.14, and an FF of 55.4% ± 0.52. Since the center point conditions were repeated across four devices, their overall performance statistics are summarized in Table 4, highlighting a low standard deviation of 0.34 and a standard error of 0.20 in PCE, indicating good reproducibility.

**Table 4:** Typical AJP Device Performance Statistics of the Center Point Devices on the BBD

**device no.**	**statistics**	***V*_oc_** [mV]	***J*_sc_** [mA/cm^2^]	**FF** [%]	**PCE** [%]
13	mean	798	24.15	53.82	10.38
	sd	2.8	0.07	1.45	0.31
14	mean	796	24.20	55.40	10.67
	sd	1.3	0.14	0.52	0.15
15	mean	798	23.29	56.32	10.46
	sd	2.0	0.20	0.87	0.19
16	mean	798	23.17	55.15	10.20
	sd	1.4	0.24	0.97	0.22
mean of means		797	23.70	55.17	10.42
standard deviation (sd)		1.77	0.88	1.41	0.34
standard error		0.9	0.4	0.7	0.2

The boxplots of the *J–V* characteristics under the different BBDoE conditions are shown in Figures S4–S6; they suggest a possible influence of the flow rate on the fill factor (FF), which, according to case 2 in Reference 32, may be linked to shunt losses. However, one-dimensional drift-diffusion simulations were performed to extract the shunt resistance values, confirming that the devices were not shunted. The response surface plots and statistical analysis of *R*_shunt_ are provided in Figures S15 and S16, respectively. Therefore, we attribute the lower FF values to morphological nonuniformities introduced during the ultrasonic aerosol jet printing of the donor material (PM6) over the spin-coated acceptor (Y12), as evidenced in Figure S1.

While our primary focus was on optimizing the sequential deposition of PM6 onto a precast Y12 film, we also fabricated a reference device using a PM6:Y12 ink deposited via uAJP for comparison. As shown in Table 5, from the PM6:Y12 ink approach, we reached a PCE of 9.29%, lower than the 10.67% PCE achieved with the optimized sequential deposition strategy; see Device 14. These results confirm the advantage of tuning the sequential deposition of a donor material via uAJP and that the sequential deposition approach leads to significantly higher efficiency, particularly due to improvements in current density and fill factor.

**Table 5:** Mean *J–V* Parameter Comparison: Mixed-Ink uAJP vs. Optimized Sequential uAJP

**parameter**	**statistics**	**PM6:Y12 ink uAJP**	**sequential uAJP**
*V*_oc_ [mV]	mean	776.6	796
	sd	1.5	1.3
*J*_sc_ [mA/cm^2^]	mean	22.43	24.20
	sd	0.09	0.14
FF [%]	mean	53.3	55.4
	sd	0.7	0.52
PCE [%]	mean	9.29	10.67
	sd	0.18	0.15

Additionally, we employed the Bayesian optimization using the optimPV python package [https://github.com/openPV-lab/optimPV][Bibr ref31] in combination with drift-diffusion (DD) simulations using SIMsalabim^33^ to extract charge transport parameters from the best-performing pixel of each inverted OPV device fabricated within the Box–Behnken design of experiments (BBDoEs). As a result of the simulations, we obtained the corresponding simulated *J–V* curves under three light intensities, 0.1, 0.5, and 1 sun, which are shown in Figure S14, together with the fitting parameter values summarized in Table S1. More discussions regarding the DD simulations can be found in the Supporting Information.

### Vertical Blending of the Active Layer

Moreover, FLAS measurements of net donor and net acceptor layers are shown in Figure S8, and there, the 0–0 transition is higher than the 0–1 transition at the top surface, implying a good molecular ordering in the donor net layer. To elucidate the vertical stacking of the active layer, we conducted FLAS and cross-sectional STEM-EELS measurements for the inverted OPV device focused on the active layer region, as shown in Figure 4. Moreover, Figure 4a–c suggests that the HOMO/LUMO levels may vary with film depth, potentially enhancing photon harvesting. However, fluctuations in charge transport levels along the film-depth direction could lead to reductions in *V*_oc_ and FF, consistent with the trends observed in Figures S4–S6. Furthermore, the ratio of the 0–0 to 0–1 transition could be associated with the crystallinity or molecular ordering of the donor, which might be depth-dependent.^7,34–36^

**Figure 4:**
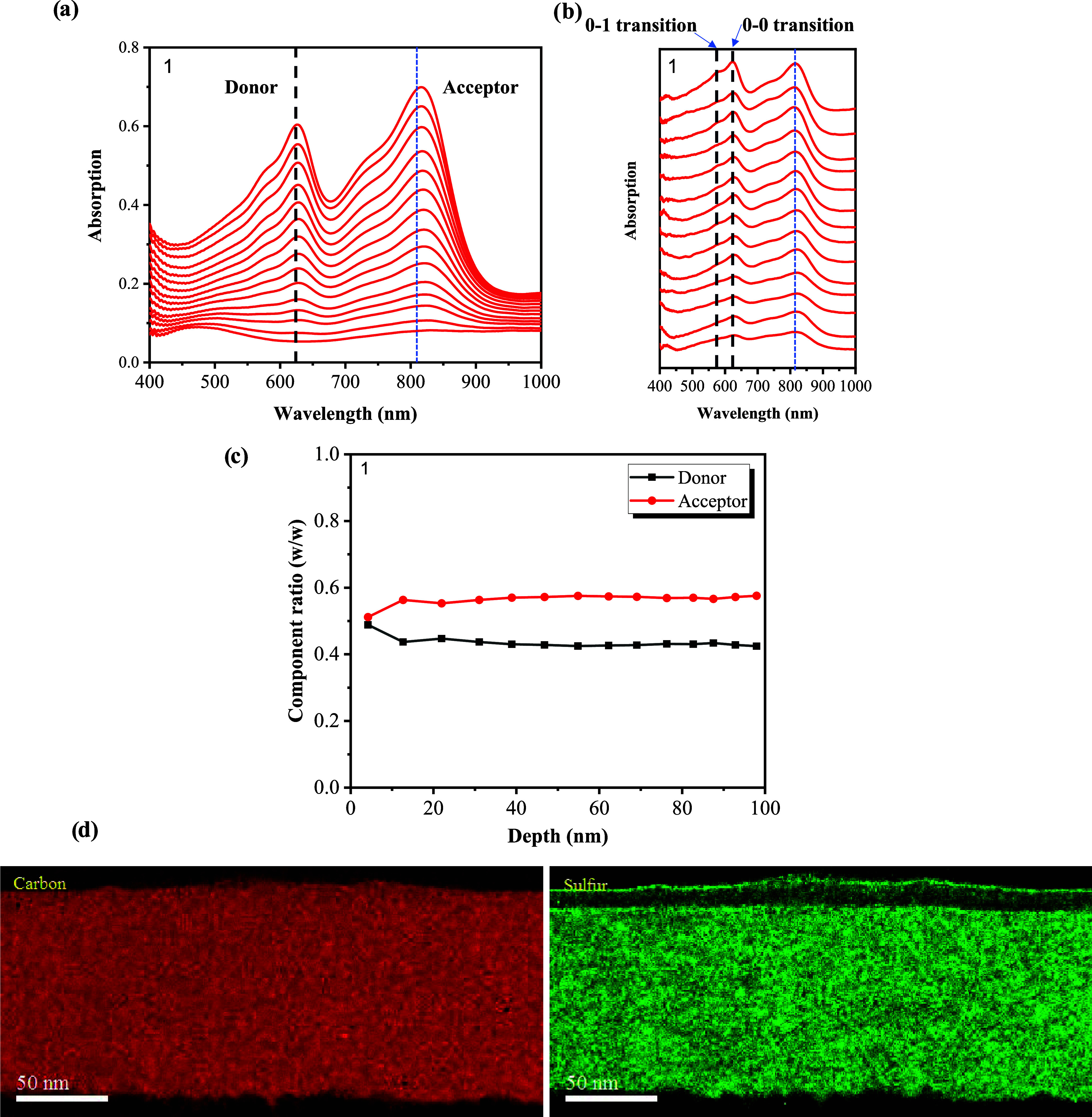
Film-depth profiling of the active layer. (a) In situ light absorption spectra at different film thicknesses over wavelength during the etching by soft plasma. (b) Film-depth-dependent light absorption spectra (FLAS) and (c) concentration (w/w) over depth as obtained from panel (a). For clarity, the spectra in panel (b) were vertically realigned, and the spectra from top to bottom represent the MoOx/active layer interface (film depth 0 nm) toward the active layer/ZnO interface (film depth 100 nm). (d) Cross-sectional STEM-EELS carbon/sulfur maps of the inverted OPV devices.

Moreover, in Figure 4d, the EELS elemental mapping of sulfur (S–K) and carbon (C–K) elucidates the spatial distribution of PM6 (sulfur-rich) and Y12 (carbon-rich). The total active layer is revealed to be ∼115 nm. It is very clear in the sulfur map that the surface topmost layer of about 10 nm displays significantly less amount of sulfur. Correspondingly, there is no clear enrichment of carbon in this thin layer, as expected for the HTL BMHTL1 used in this sample. In the active layer, only a very slight elemental enrichment in sulfur on the scale of about 20–30 nm can be revealed. This type of largely homogeneous distribution of both elements indicates that the layer exhibits a BHJ vertical composition profile, which is distinctly different compared to a bilayer structure, as studied earlier.^12^ Both FLAS and cross-sectional STEM-EELS results suggest that the active layer exhibits a BHJ vertical phase distribution. This observation is consistent with the study by Zhan et al., which reported that normal-structure OPV devices processed using LbL deposition with a binary blend (PM6:BO-4Cl) exhibited a BHJ-like morphology, whereas in LbL-processed ternary blends, the reduced miscibility of BTP-S2 with PM6 promoted vertical phase separation.^11^

Additionally, complementary morphological analyses, including GIWAXS, were performed on a device fabricated under the center point conditions of the Box–Behnken design of experiments. Measurements were taken at two distinct regions of the device, one consisting of a Y12/ZnO/ITO/glass stack and the other containing an additional PM6 layer on top, forming PM6/Y12/ZnO/ITO/glass stack. In Figure 5a, a 2D GIWAXS measurement of the first region with a neat Y12 thin film is displayed. It exhibits numerous scattering peaks originating from highly ordered Y12 with long-range order. Corresponding cake cuts extracted from this measurement are also displayed in Figure 5c, bottom. Following the deposition of the Y12 layer, PM6 was applied via aerosol jet printing (AJP) and the GIWAXS measurement of the second region, which contains both PM6 and Y12, is shown in Figure 5b, and its corresponding cake cuts are displayed in Figure 5c, top. The incident angle of the X-ray beam is well beyond the critical angle so that the full depth of the thin film is probed (the substrate signal is visible at large q, not displayed here).

**Figure 5:**
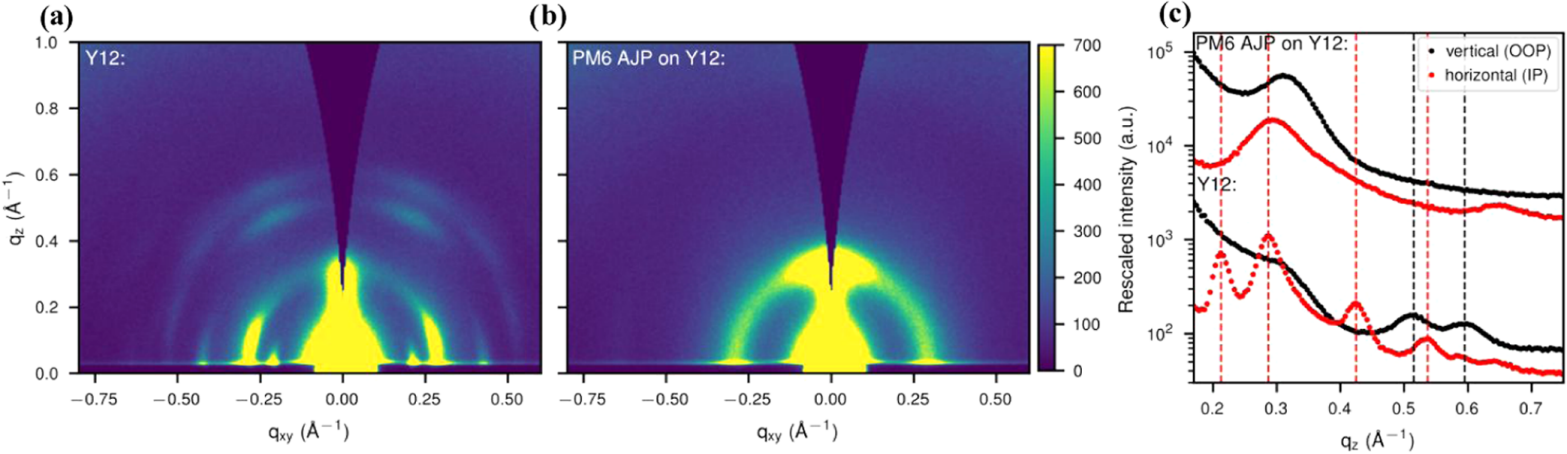
2D GIWAXS measurements of (a) a neat Y12 film spin-coated from o-xylene and (b) a thin film, where additionally PM6 was deposited onto the Y12 film by aerosol jet printing. (c) Comparison of cake cuts extracted from both measurements in the horizontal (in-plane, IP) and vertical (out-of-plane, OOP) directions. The positions of the neat Y12 peaks are indicated by vertical dashed lines (black: vertical, red: horizontal). The cuts are shifted for clarity for the combined sample by the same factor.

The comparison of the two 2D GIWAXS measurements and the cake cuts shows that numerous scattering features from the neat Y12 are missing in the blend thin film (e.g., at about 0.21, 0.42, and 0.54 Å^–1^ in the horizontal and at about 0.52 and 0.60 Å^–1^ in the vertical). Instead, a strong first-order lamellar scattering is observed at about 0.31 Å^–1^ in the vertical direction originating from PM6 with an additional azimuthal distribution. Higher orders are not observed but a weak backbone scattering peak in the horizontal direction (0.65 Å^–1^), indicating short-range order only for PM6. The dominant lamellar scattering feature of Y12 in the horizontal direction at about 0.29 Å^–1^ is also present in the blend thin film but is significantly broadened. Overall, the presence of just one strongly broadened scattering feature of Y12, while all other scattering features vanish, demonstrates that the long-range ordering of the neat Y12 film is lost once PM6 is deposited on top.

This sequential processing raises the question of whether the resulting morphology is a bilayer comprising separate Y12 and PM6 layers or a bulk heterojunction (BHJ) characterized by phase intermixing. A BHJ structure is suggested by the results of GIWAXS. If the blend thin film was a bilayer without intermixing of Y12 and PM6, the nanostructure of Y12 would not be affected by PM6, and a superposition of the neat Y12 and additional PM6 GIWAXS patterns would be expected. However, this is not the case, although we probed the full depth of the film. Hence, the Y12 must be partially intermixed with the PM6 (i.e., in a BHJ), leading to the vanishing long-range ordering of the Y12 observed in the GIWAXS measurements. The GIWAXS results from the thin film, where PM6 was deposited by AJP on top of the Y12 thin film, are also highly comparable to thin films deposited in a one-step process from blend solutions of a polymer donor and a nonfullerene acceptor, which is known to form a BHJ.^37–40^

The observed BHJ morphology, despite sequential deposition, is likely caused by partial infiltration or mixing of the donor material (PM6) into the loosely packed, spin-coated Y12 acceptor layer. This can be attributed to the PM6 flexible backbone, its high solubility in o-xylene, and the fine droplets produced during uAJP, which may penetrate or partially diffuse into the underlying layer during deposition. To mitigate this, potential strategies for future research could include modifying the donor solvent system, enhancing thermal annealing or crystallization of the acceptor prior to donor deposition by using higher temperature or time or by using a cross-linker on the acceptor layer, or introducing a thin interfacial buffer layer.^13,14^

### Stability Test of Inverted OPV Devices

The *J–V* characteristics of the stability test performed on the inverted OPV devices are shown in Figure 6, while the normalized PCE values, similar to those reported elsewhere^41^ after 1080 h of continuous one-sun white LED illumination, are presented in Figure S10. Device no. 10, performed with 80 sccm, 0.475 A, and 12 mm/s conditions, showed the lowest PCE values, before and after the stability test. Additionally, device no. 5 performed at 80 sccm, 0.45 A, and 10 mm/s also presented low efficiency before and after the stability test. Both devices had in common the lowest level for flow, which might indicate that low flows might be related to low stabilities. Interestingly, Device no. 7, fabricated at 120 sccm, 0.45 A, and 10 mm/s, showed a PCE nearly matching the highest value obtained at center point conditions. Fascinatingly, its PCE increased during degradation, likely due to light soaking, where illumination enhances donor–acceptor organization and interfacial contact, temporarily improving performance.^42,43^ Encouragingly, devices with a higher initial PCE also demonstrated better stability, suggesting a strong link between efficiency and long-term performance under center point conditions.

**Figure 6:**
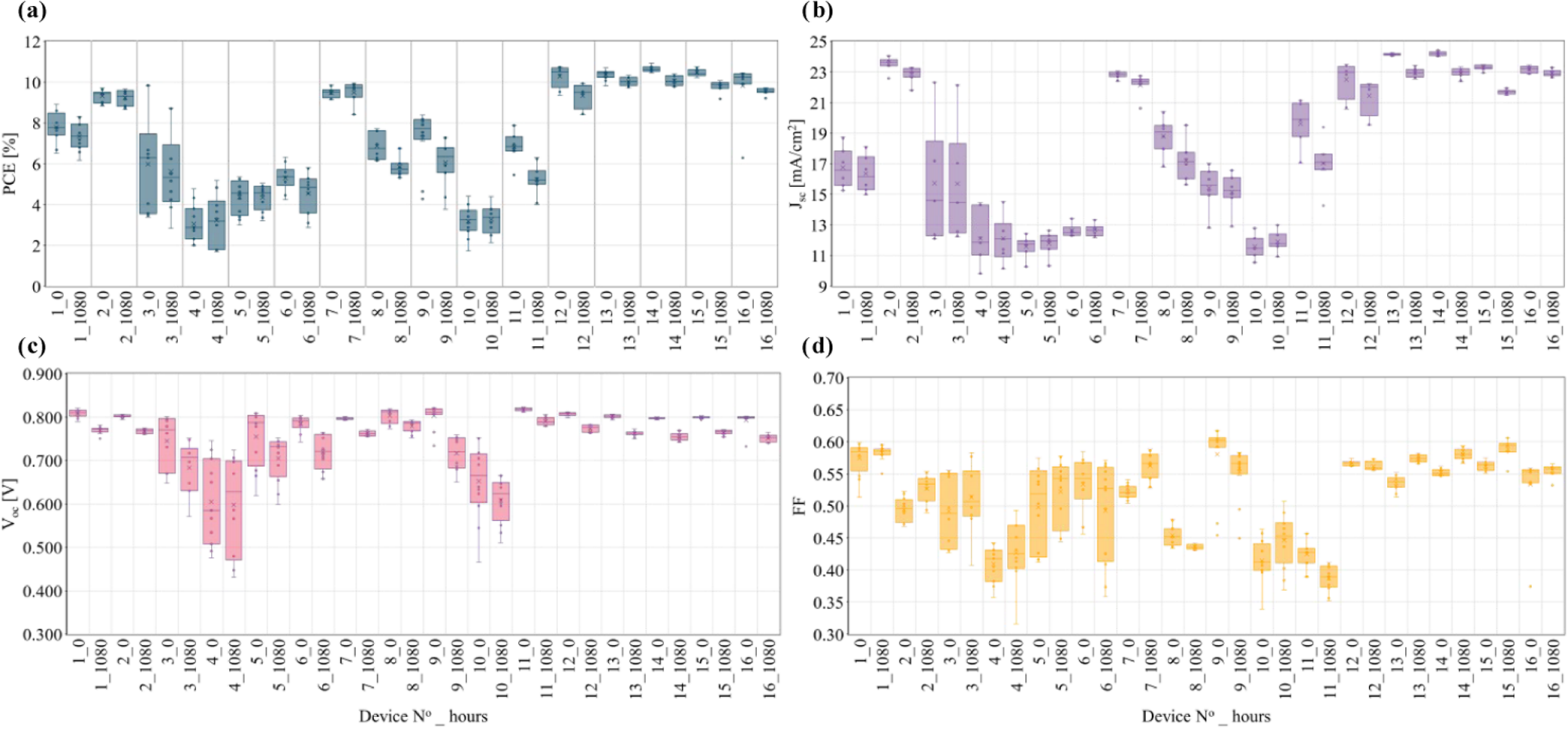
*J–V* characteristic parameters before (0) and after 1080 h of stability test performed under one-sun illumination with white light LED on the inverted OPV devices with the uAJP donor material and measured under AM1.5 G in LineOne: (a) PCE, (b) *J*_sc_, (c) *V*_oc_, and (d) FF.

## Conclusions

We have successfully demonstrated a novel method to tune the donor-to-acceptor ratio and PCE in inverted OPV devices by sequentially depositing the donor via ultrasonic aerosol jet printing (uAJP) onto a spin-coated acceptor layer. Despite sequential deposition, FLAS and cross-sectional TEM measurements revealed a BHJ vertical phase distribution within the active layer under the tested conditions. Using the Box–Behnken design of experiments (DoEs), we systematically investigated the influence of uAJP factors, such as speed, power, and flow, and identified nonlinear effects on both the donor/acceptor ratio and the power conversion efficiency (PCE) of the inverted OPV devices. The optimal PCE values were achieved at the center point conditions, i.e., 100 sccm flow rate, 10 mm/s speed, and 0.475 A power, while the low level of flow led to poor efficiency and stability, probably due to insufficient donor material deposition. These findings highlight the importance of fine-tuning deposition conditions while ultrasonic aerosol jet printing the donor material to balance both efficiency and stability, offering a pathway for high-throughput fabrication of inverted OPV devices at the laboratory scale. Further studies, such as research on different material systems for the active layer, or a deeper morphological characterization such as GIWAXs or STEM-EELS to confirm BHJ morphology at all of the conditions of the BBDoE, or even machine learning to predict optimal processing conditions perhaps with fewer experiments, might accelerate the fabrication of efficient and stable inverted OPV devices.

## Supplementary Material



## Notes

Photographs of the 16 inverted OPV devices of the BBDoE (Figure S1); response surface method with the Box–Behnken design of experiments using ratio as the control variable; left side: response surface plots, and right side: contour plots; screening two factors of (a) flow and power, (b) flow and speed, and (c) power and speed, while holding the third factor at its respective mean value (Figure S2); response surface method with the Box–Behnken design of experiments using *J*_sc_ as the control variable; left side: response surface plots, and right side: contour plots; screening two factors of (a) flow and power, (b) flow and speed, and (c) power and speed, while holding the third factor at its respective mean value (Figure S3); *J–V* characteristic under AM1.5G of the OPV devices based on the factor flow of the donor while screening the (a) speed, (b) power factors from ultrasonic aerosol jet printing the donor material (Figure S4); *J–V* characteristic under AM1.5G of the OPV devices based on the factor power of the donor while screening the (a) speed and (b) flow from the ultrasonic aerosol jet printing the donor material (Figure S5); *J–V* characteristic under AM1.5G of the OPV devices based on the factor speed of the donor while screening the (a) power and (b) flow factors from ultrasonic aerosol jet printing the donor material (Figure S6); *J–V* curves of the OPV devices based on the factors from left to right flow, power and speed under (a) AM1.5G and (b) in the dark (Figure S7); FLAS measurement: absorption at different film thicknesses over wavelength and close of (a) net donor layer and (b) net acceptor layer (Figure S8); measured thickness of uAJP donor films printed under the same BBD conditions over plain glass versus the OD donor peak intensity (Figure S9); normalized PCE and FoMse under one-sun illumination before and after 1080 h degradation on (a) numbers 1–8 and (b) numbers 9–16 inverted OPV devices with ultrasonic aerosol jet printed donor material (Figure S10); statistical analysis of ratio as the control variable on the BBD (Figure S11); and statistical analysis of PCE as the control variable on the BBD (Figure S12) (PDF)

## Abbreviations

OPV: organic photovoltaic devices
uAJP: ultrasonic aerosol jet printing
BBDoEs: Box–Behnken design of experiments
LbL: layer-by-layer
EELS: electron energy-loss spectroscopy
STEM: scanning transmission electron microscope

## References

[1] Li F., Lin F. R., Jen A. K. Y. (2024). Current State and Future Perspectives
of Printable Organic and Perovskite Solar Cells. Adv. Mater..

[2] Chang K., Yu B., Liu L., Fang D., Zhao X., Mi B., Huang W., Deng W. (2023). Efficient Fully-Sprayed Organic Solar
Cells with Coffee-Ring-Free Photoactive Layer and Alloy Top-Electrode. Adv. Mater. Technol..

[3] Fiter L., Mustafa M. N., Sulaiman Y. (2023). Optimization
of Power Conversion
Efficiency of BaTiO3 as a Compact Layer in DSSC Using Response Surface
Methodology/Box-Behnken Design. Optik (Stuttg)..

[4] Brabec C. J., Hauch J. A., Schilinsky P., Waldauf C. (2005). Production Aspects
of Organic Photovoltaics and Their Impact on the Commercialization
of Devices. MRS Bull..

[5] Xiao M., Meng Y., Tang L., Li P., Tang L., Zhang W., Hu B., Yi F., Jia T., Cao J., Xu C., Lu G., Hao X., Ma W., Fan Q. (2023). Xiao - Solid Additive-Assisted Selective Optimization
Strategy for
Sequential Deposited Active.Pdf.. Adv. Funct
Materials.

[6] Xia R., Brabec C. J., Yip H. L., Cao Y. (2019). High-Throughput Optical
Screening for Efficient Semitransparent Organic Solar Cells. Joule.

[7] Shen Z., Yu J., Lu G., Wu K., Wang Q., Bu L., Liu X., Zhu Y., Lu G. (2023). Surface Crystallinity Enhancement
in Organic Solar Cells Induced by Spinodal Demixing of Acceptors and
Additives. Energy Environ. Sci..

[8] Chang B., Jiang B. H., Chen C. P., Chen K., Chen B. H., Tan S., Lu T. C., Tsao C. S., Su Y. W., Yang S. Da., Chen C. S., Wei K. H. (2024). Achieving High Efficiency and Stability
in Organic Photovoltaics with a Nanometer-Scale Twin p-i-n Structured
Active Layer. ACS Appl. Mater. Interfaces.

[9] Wang H.-C., Cheng P., Tan S., Chen C.-H., Chang B., Tsao C.-S., Chen L.-Y., Hsieh C.-A., Lin Y.-C., Cheng H.-W., Yang Y., Wei K.-H. (2021). Sequential Deposition
of Donor and Acceptor Provides High-Performance.Pdf. Adv. Energy Mater..

[10] Sun R., Guo J., Wu Q., Zhang Z., Yang W., Guo J., Shi M., Zhang Y., Kahmann S., Ye L., Jiao X., Loi M. A., Shen Q., Ade H., Tang W., Brabec C. J., Min J. (2019). A Multi-Objective Optimization-Based
Layer-by-Layer Blade-Coating Approach for Organic Solar Cells: Rational
Control of Vertical Stratification for High Performance. Energy Environ. Sci..

[11] Zhan L., Li S., Xia X., Li Y., Lu X., Zuo L., Shi M., Chen H. (2021). Layer-by-Layer Processed Ternary Organic Photovoltaics
with Efficiency over 18%. Adv. Mater..

[12] Wang R., Jiang Y., Gruber W., He Y., Wu M., Weitz P., Zhang K., Lüer L., Forberich K., Unruh T., Spiecker E., Deibel C., Li N., Brabec C. J. (2022). Tailoring the Nature of Interface States in Efficient
and Stable Bilayer Organic Solar Cells by a Transfer-Printing Technique. Adv. Mater. Interfaces.

[13] Sampaio P. G. V., Orestes Aguirre González M., de Oliveira Ferreira P., da Cunha Jácome Vidal P., Pinheiro Pereira J. P., Rodrigues Ferreira H., Oprime P. C. (2020). Overview of Printing
and Coating
Techniques in the Production of Organic Photovoltaic Cells. Int. J. Energy Res..

[14] Ju Z., Lv R., Ansari A. A., Lin J. (2025). Recent Advances in Additive Manufacturing
for Solar Cell Based on Organic/Inorganic Hybrid Materials. InfoMat.

[15] Nowak-Król A., Shoyama K., Stolte M., Würthner F. (2018). Naphthalene
and Perylene Diimides-Better Alternatives to Fullerenes for Organic
Electronics?. Chem. Commun..

[16] Arango-Marín V., Rocha-Ortiz J. S., Osterrieder T., Barabash A., Osvet A., Wortmann J., Heumüller T., Liu C., Hauch J., Brabec C. J. (2025). Aerosol-Jet-Printed
Silver Nanowires as Top Electrodes
in Organic Photovoltaic Devices. Sol. RRL.

[17] Zhang J., Liu B., Liu Z., Wu J., Arnold S., Shi H., Osterrieder T., Hauch J. A., Wu Z., Luo J., Wagner J., Berger C. G., Stubhan T., Schmitt F., Zhang K., Sytnyk M., Heumueller T., Sutter-Fella C. M., Peters I. M., Zhao Y., Brabec C. J. (2023). Optimizing
Perovskite Thin-Film Parameter Spaces with Machine Learning-Guided
Robotic Platform for High-Performance Perovskite Solar Cells. Adv. Energy Mater..

[18] Wagner J., Berger C. G., Du X., Stubhan T., Hauch J. A., Brabec C. J. (2021). The Evolution of Materials Acceleration Platforms:
Toward the Laboratory of the Future with AMANDA. J. Mater. Sci..

[19] Osterrieder T., Schmitt F., Lüer L., Wagner J., Heumüller T., Hauch J., Brabec C. J. (2023). Autonomous
Optimization of an Organic
Solar Cell in a 4-Dimensional Parameter Space. Energy Environ. Sci..

[20] Weitz P., Le Corre V. M., Du X., Forberich K., Deibel C., Brabec C. J., Heumüller T. (2023). Revealing
Photodegradation Pathways of Organic Solar Cells by Spectrally Resolved
Accelerated Lifetime Analysis. Adv. Energy Mater..

[21] Seiberlich M., Strobel N., Ruiz-Preciado L. A., Ruscello M., Lemmer U., Hernandez-Sosa G. (2021). Aerosol-Jet-Printed Donor-Blocking Layer for Organic
Photodiodes. Adv. Electron. Mater..

[22] Gamba L., Johnson Z. T., Atterberg J., Diaz-Arauzo S., Downing J. R., Claussen J. C., Hersam M. C., Secor E. B. (2023). Systematic
Design of a Graphene Ink Formulation for Aerosol Jet Printing. ACS Appl. Mater. Interfaces.

[23] Sherman D. A., Landberg E., Peringath A. R., Kar-Narayan S., Tan J. C. (2024). Fine-Scale Aerosol-Jet Printing of
Luminescent Metal-Organic
Framework Nanosheets. ACS Appl. Mater. Interfaces.

[24] Yang P., Zhai T., Yu B., Du G., Mi B., Zhao X., Deng W. (2021). Toward All Aerosol
Printing of High-Efficiency
Organic Solar Cells Using Environmentally Friendly Solvents in Ambient
Air. J. Mater. Chem. A.

[25] Basu R., Siah K. S., Distler A., Häußler F., Franke J., Brabec C. J., Egelhaaf H. J. (2023). Aerosol-Jet-Printed
Encapsulation of Organic Photovoltaics. Adv.
Eng. Mater..

[26] Eckstein R., Hernandez-Sosa G., Lemmer U., Mechau N. (2014). Aerosol Jet Printed
Top Grids for Organic Optoelectronic Devices. Org. Electron..

[27] Kopola P., Zimmermann B., Filipovic A., Schleiermacher H.-F., Greulich J., Rousu S., Hast J., Myllylä R., Würfel U. (2012). Aerosol Jet
Printed Grid for ITO-Free Inverted Organic
Solar Cells. Sol. Energy Mater. Sol. Cells.

[28] Yang C., Zhou E., Miyanishi S., Hashimoto K., Tajima K. (2011). Preparation of Active Layers in Polymer
Solar Cells
by Aerosol Jet Printing. ACS Appl. Mater. Interfaces.

[29] Steirer K.
X., Reese M. O., Rupert B. L., Kopidakis N., Olson D. C., Collins R. T., Ginley D. S. (2009). Ultrasonic Spray
Deposition for Production of Organic Solar Cells. Sol. Energy Mater. Sol. Cells.

[30] Spooner E. L. K., Cassella E. J., Smith J. A., Catley T. E., Burholt S., Lidzey D. G. (2023). Air-Knife-Assisted
Spray Coating of Organic Solar Cells. ACS Appl.
Mater. Interfaces.

[31] Wagner M., Distler A., Le Corre V. M., Zapf S., Baydar B., Schmidt H. D., Heyder M., Forberich K., Lüer L., Brabec C. J., Egelhaaf H. J. (2023). Cutting
“Lab-to-Fab”
Short: High Throughput Optimization and Process Assessment in Roll-to-Roll
Slot Die Coating of Printed Photovoltaics. Energy
Environ. Sci..

[32] These A., Koster L. J. A., Brabec C. J., Le Corre V. M. (2024). Beginner’s
Guide to Visual Analysis of Perovskite and Organic Solar Cell Current
Density–Voltage Characteristics. Adv.
Energy Mater..

[33] Koopmans M., Corre V., Koster L. (2022). SIMsalabim: An Open-Source Drift-Diffusion
Simulator for Semiconductor Devices. J. Open
Source Softw..

[34] Lu G., Shen Z., Wang H., Bu L., Lu G. (2023). Optical Interference
on the Measurement of Film-Depth-Dependent Light Absorption Spectroscopy
and a Correction Approach. Rev. Sci. Instrum..

[35] Bu L., Hu M., Lu W., Wang Z., Lu G. (2018). Printing Semiconductor–Insulator
Polymer Bilayers for High-Performance Coplanar Field-Effect Transistors. Adv. Mater..

[36] Yu J., Xing Y., Shen Z., Zhu Y., Neher D., Koch N., Lu G. (2021). Infrared Spectroscopy Depth Profiling
of Organic Thin Films. Mater. Horizons.

[37] Eller F., McNeill C. R., Herzig E. M. (2024). Tackling
P3HT:Y-Series Miscibility
Through Advanced Processing for Tunable Aggregation. Adv. Energy Mater..

[38] Perdigón-Toro L., Phuong L. Q., Eller F., Freychet G., Saglamkaya E., Khan J. I., Wei Q., Zeiske S., Kroh D., Wedler S., Köhler A., Armin A., Laquai F., Herzig E. M., Zou Y., Shoaee S., Neher D. (2022). Understanding
the Role of Order in Y-Series Non-Fullerene Solar Cells to Realize
High Open-Circuit Voltages. Adv. Energy Mater..

[39] Kroh D., Eller F., Schötz K., Wedler S., Perdigón-Toro L., Freychet G., Wei Q., Dörr M., Jones D., Zou Y., Herzig E. M., Neher D., Köhler A. (2022). Identifying the Signatures of Intermolecular Interactions
in Blends of PM6 with Y6 and N4 Using Absorption Spectroscopy. Adv. Funct. Mater..

[40] Di
Mario L., Garcia Romero D., Pieters M. J., Eller F., Zhu C., Bongiovanni G., Herzig E. M., Mura A., Loi M. A. (2023). Effects
of the Diphenyl Ether Additive in Halogen-Free Processed Non-Fullerene
Acceptor Organic Solar Cells. J. Mater. Chem.
A.

[41] Li N., McCulloch I., Brabec C. J. (2018). Analyzing the Efficiency, Stability
and Cost Potential for Fullerene-Free Organic Photovoltaics in One
Figure of Merit. Energy Environ. Sci..

[42] Weitz P., Wortmann J., Liu C., Wen T. J., Li C. Z., Heumüller T., Brabec C. J. (2024). Photodegradation of Organic Solar
Cells under Visible Light and the Crucial Influence of Its Spectral
Composition. ACS Appl. Mater. Interfaces.

[43] van
der Pol T. P. A., van Gorkom B. T., van Geel W. F. M., Littmann J., Wienk M. M., Janssen R. A. J. (2023). Origin, Nature, and Location of Defects
in PM6:Y6 Organic Solar Cells. Adv. Energy Mater..

