# Supplementary material for: Fine-Tuning Donor Material Deposition with Ultrasonic Aerosol Jet Printing to Balance Efficiency and Stability in Inverted Organic Photovoltaic Devices
Source: ACS Appl Mater Interfaces. 2025 Jul 31;17(32):46149–60. doi: 10.1021/acsami.5c09318 (PMC12356527; doi:10.1021/acsami.5c09318)
Supplement: Supplementary file 1 [file am5c09318_si_001.pdf]

Supporting Information

Fine-tuning Donor Material Deposition with Ultrasonic Aerosol Jet Printing to Balance Efficiency and Stability in Inverted Organic Photovoltaic Devices

AUTHORS: *Vanessa Arango-Marín[a,b]*, Jonas Wortmann[a,b], Tobias Osterrieder[a,b], Paul Weitz[b], Juan S. Rocha-Ortiz[a,b], Mingjian Wu[d], Xin Zhou[d], Fabian Eller[e], Thomas Heumüller[a,b], Jens A. Hauch[a,b], Chao Liu[a,b], Vincent M. Le Corre[c], Erdmann Spiecker[d], Eva M. Herzig[e], Guanghao Lu[f], Larry Lüer[a,b] and Christoph J. Brabec[a,b]**

AFFILIATIONS:

a) Department of High Throughput Methods in Photovoltaics, Forschungszentrum Jülich GmbH, Helmholtz-Institute Erlangen-Nürnberg (HI ERN), Immerwahrstraße 2, 91058 Erlangen, Germany

b) Department of Materials Science and Engineering, Institute of Materials for Electronics and Energy Technology (i-MEET), Friedrich-Alexander-Universität Erlangen-Nürnberg, Martensstraße 7, 91058 Erlangen, Germany

c) Centre for Advanced Photovoltaics and Thin-film Energy Devices (CAPE), University of Southern Denmark, Alsion 2, DK-6400 Sønderborg, Denmark

d) Institute of Micro- and Nanostructure Research (IMN) & Center for Nanoanalysis and Electron Microscopy (CENEM), Interdisciplinary Center for Nanostructured Films (IZNF), Cauerstraße 3, 91058 Erlangen, Germany

e) Dynamics and Structure Formation – Herzig Group, University of Bayreuth, Universitätsstraße 30, 95447 Bayreuth, Germany

f) Institute of Science and Technology, Xi'an Jiaotong University, Xi'an, 710054 China

E-mail: vanessa.arango@fau.de, christoph.brabec@fau.de

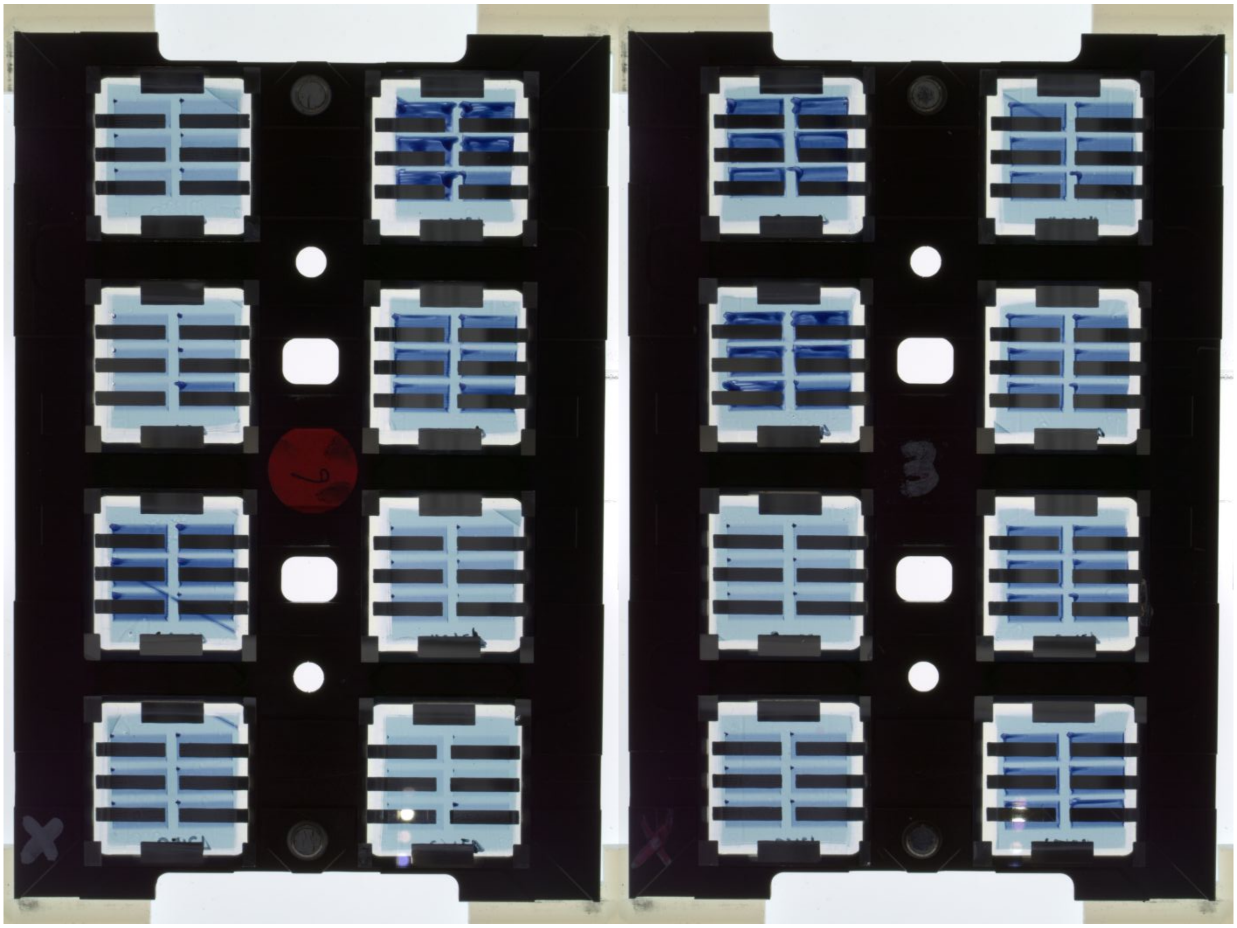

Figure S 1. Photographs of the 16 inverted OPV devices of the BBDoE

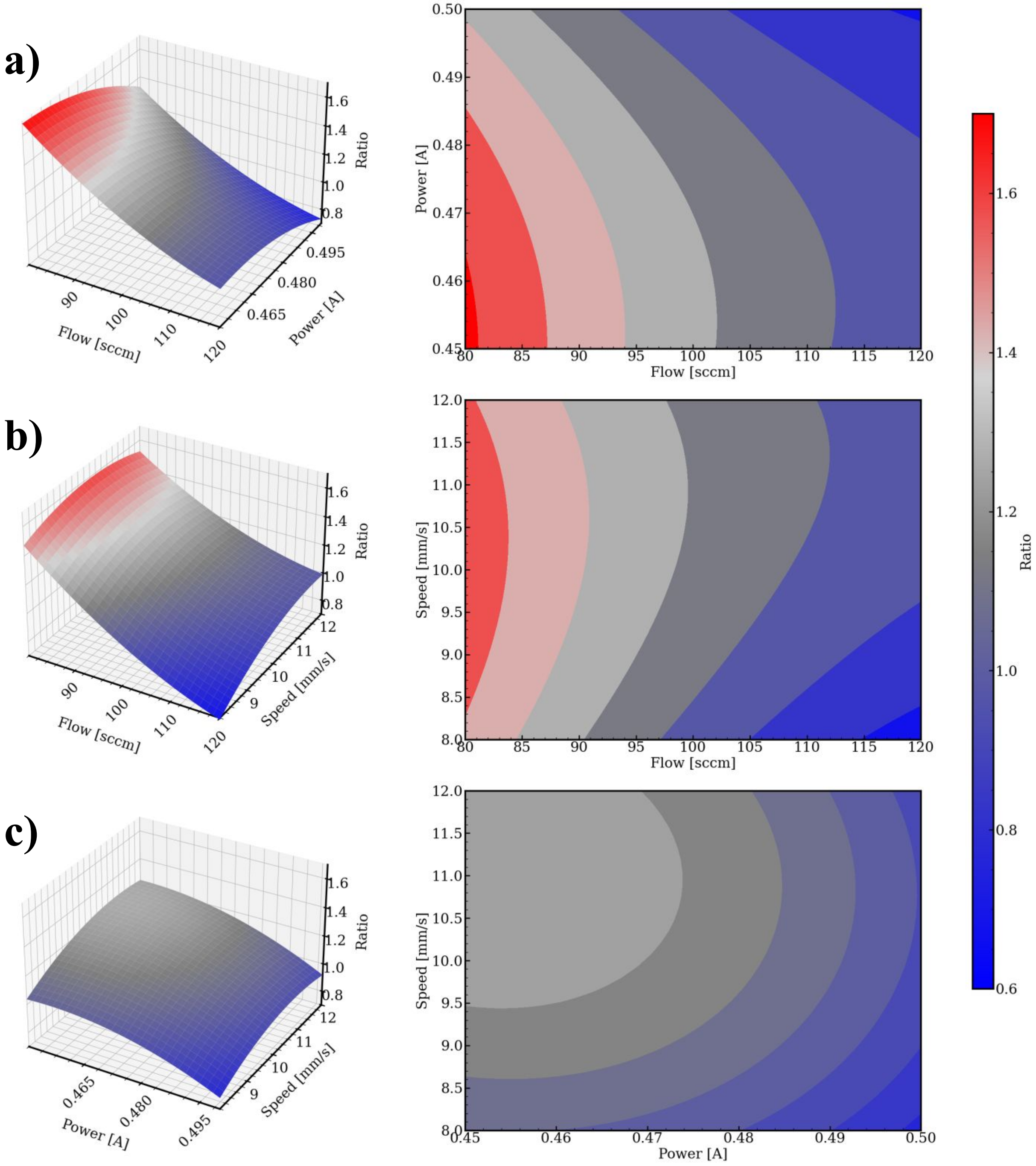

Figure S 2. Response surface method with Box-Behnken design of experiments using Ratio as the control variable. Left side: Response surface plots, and right side: Contour plots. Screening two factors of a) flow and power, b) flow and speed and c) power and speed; while holding the third factor at its respective mean value.

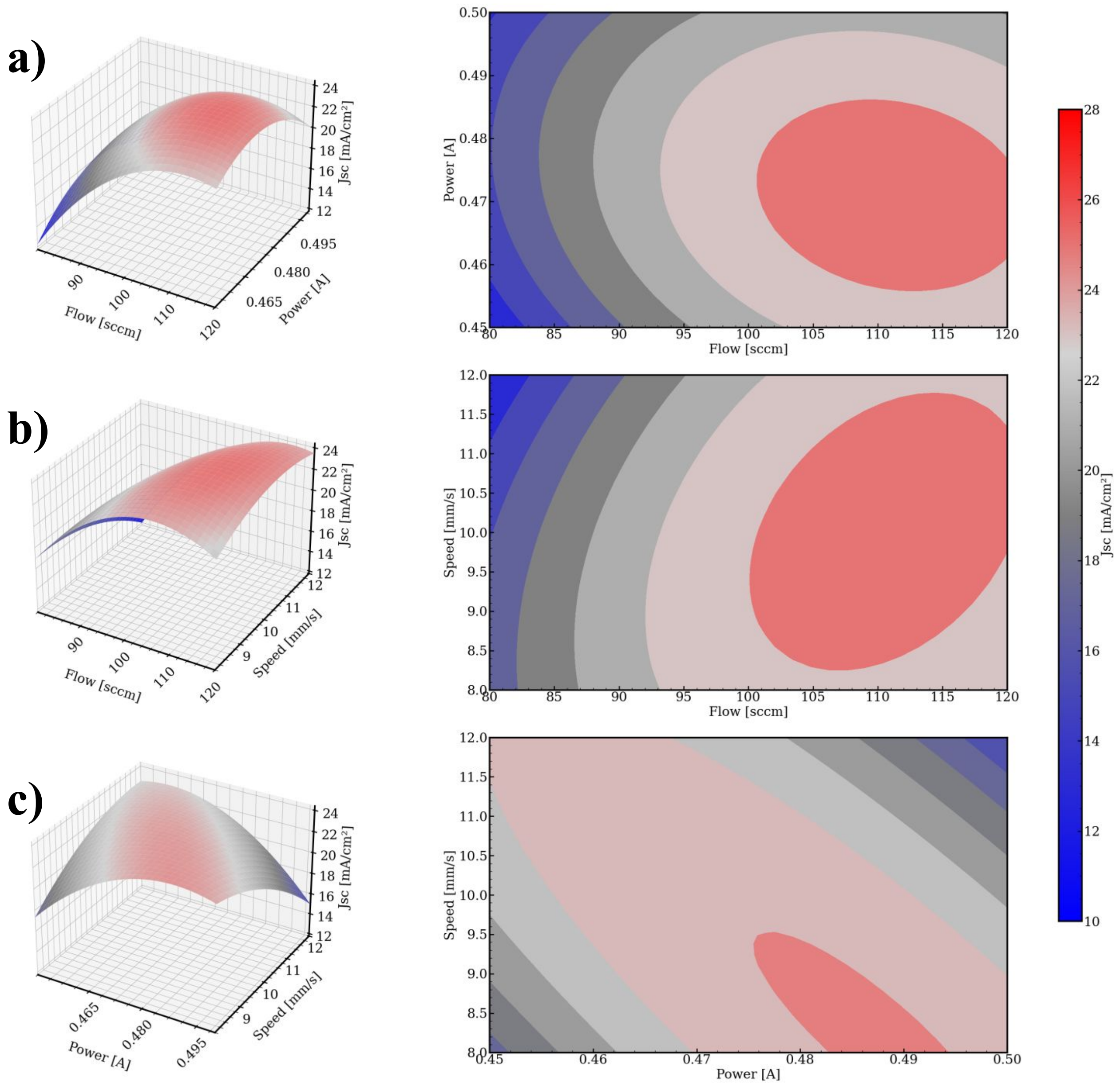

Figure S 3. Response surface method with Box-Behnken design of experiments using Jsc as the control variable. Left side: Response surface plots, and right side: Contour plots. Screening two factors of a) flow and power, b) flow and speed and c) power and speed; while holding the third factor at its respective mean value.

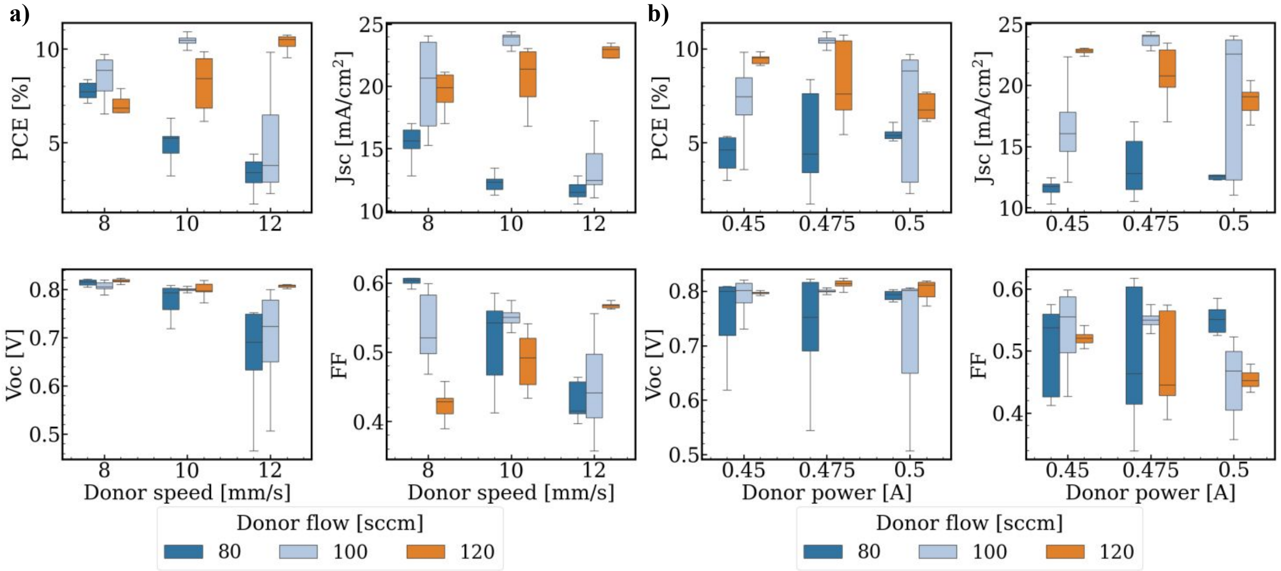

Figure S 4. JV characteristic under AM1.5G of the OPV devices based on the factor flow of the donor while screening the a) speed, b) power factors from ultrasonic aerosol jet printing the donor material.

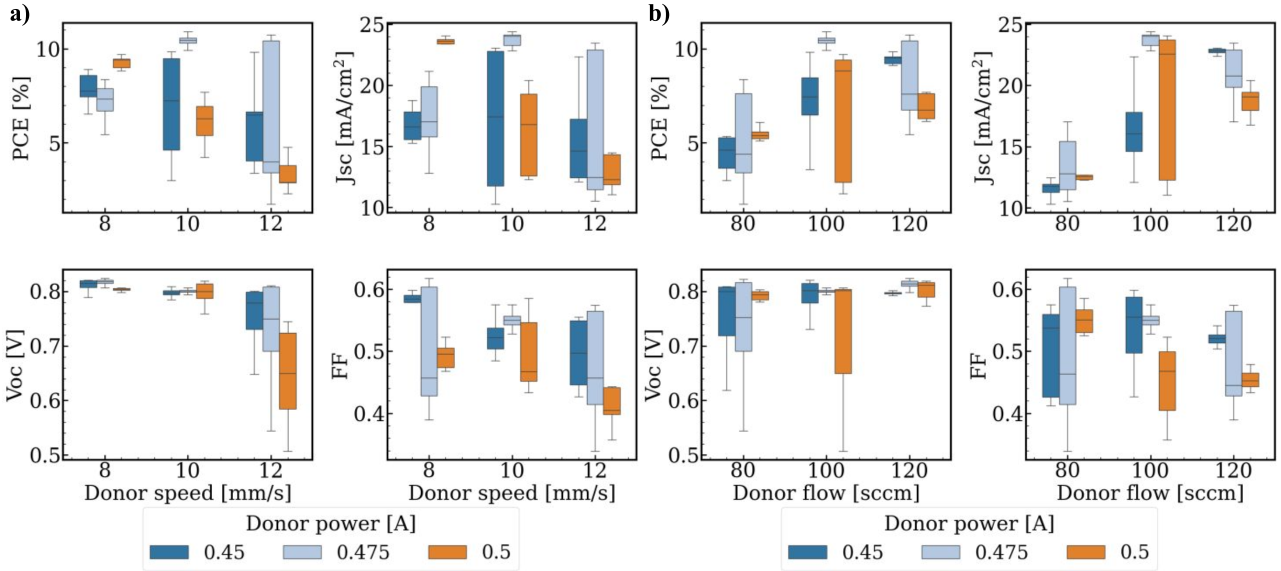

Figure S 5. JV characteristic under AM1.5G of the OPV devices based on the factor power of the donor while screening the a) speed, and b) flow from ultrasonic aerosol jet printing the donor material.

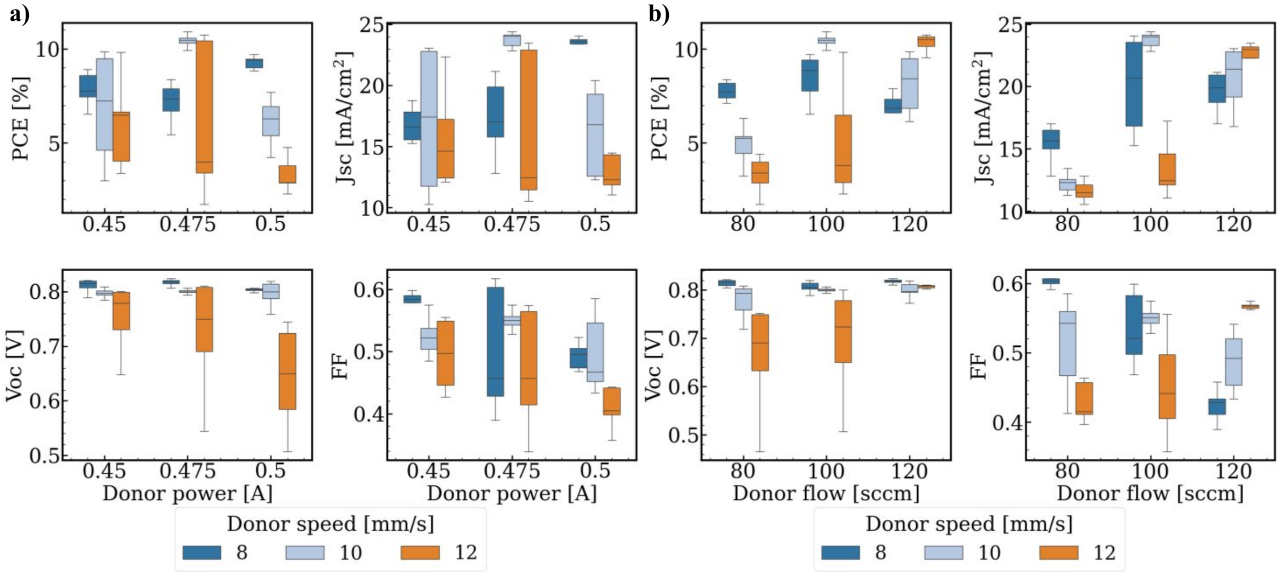

Figure S 6. JV characteristic under AM1.5G of the OPV devices based on the factor speed of the donor while screening the a) power, and b) flow factors from ultrasonic aerosol jet printing the donor material.

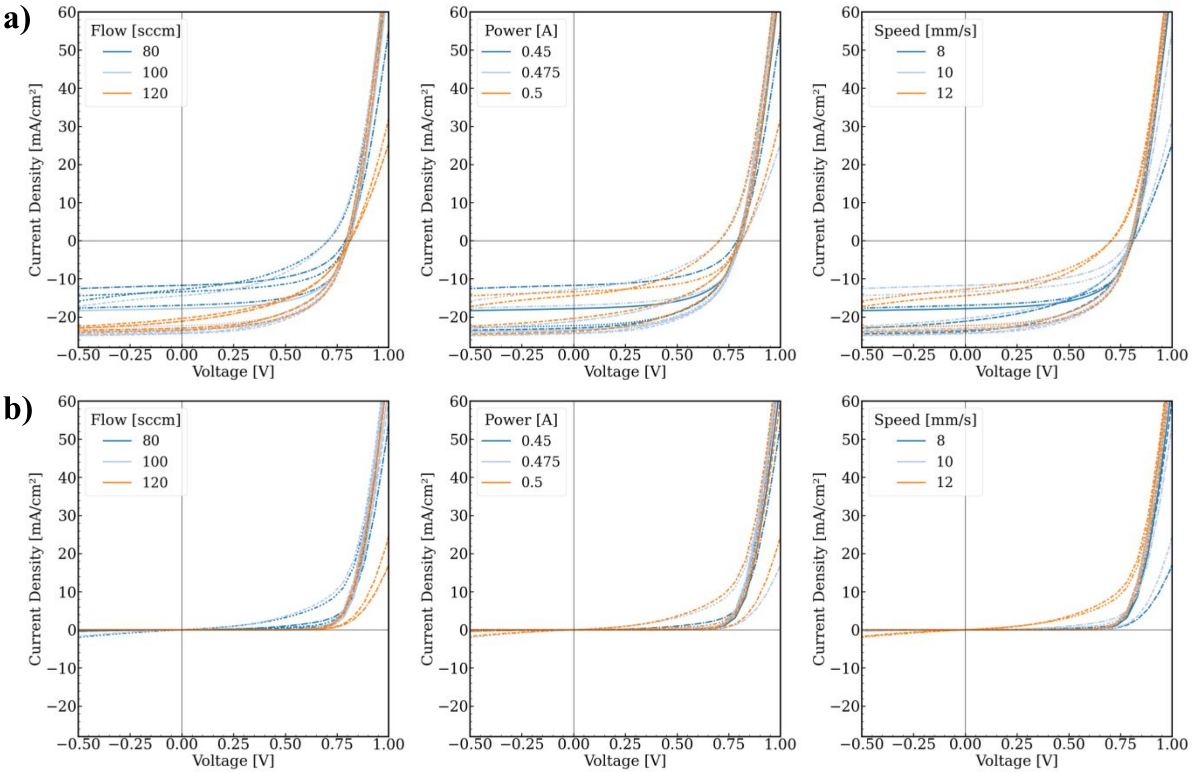

Figure S 7. JV curves of the OPV devices based on the factors from left to right flow, power and speed under a) AM1.5G and b) in the dark.

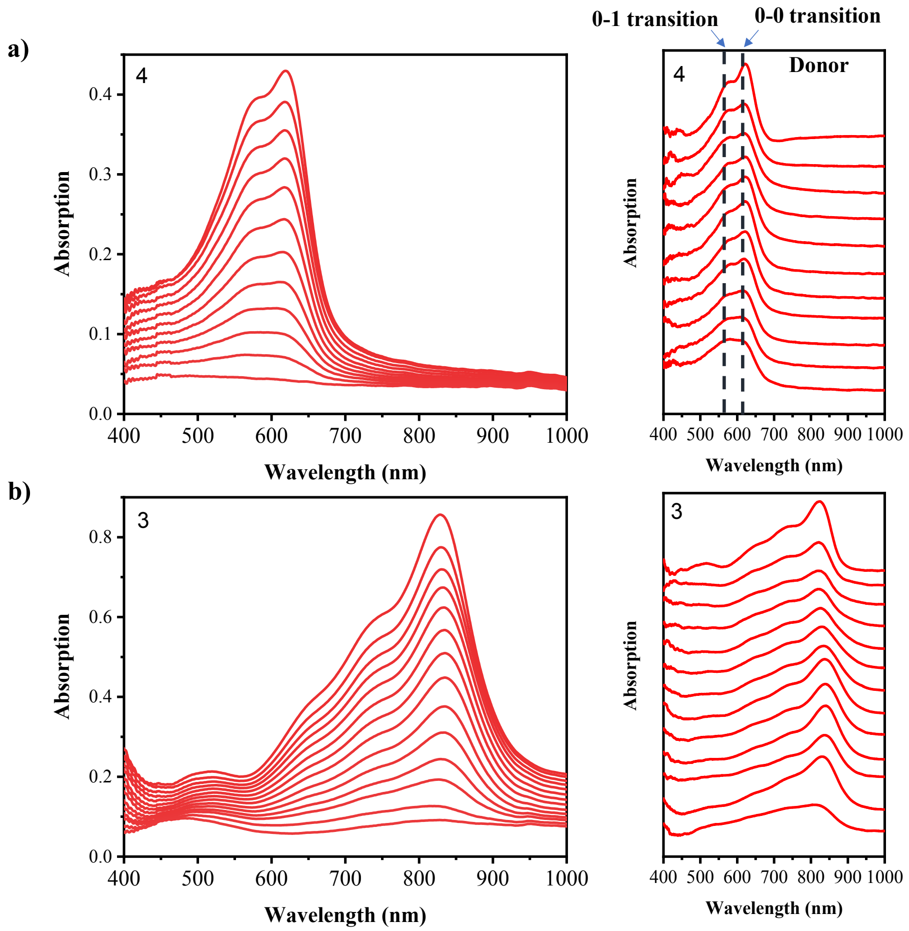

Figure S 8. FLAS measurement: Absorption at different film thicknesses over wavelength and close of a) net donor layer and b) net acceptor layer.

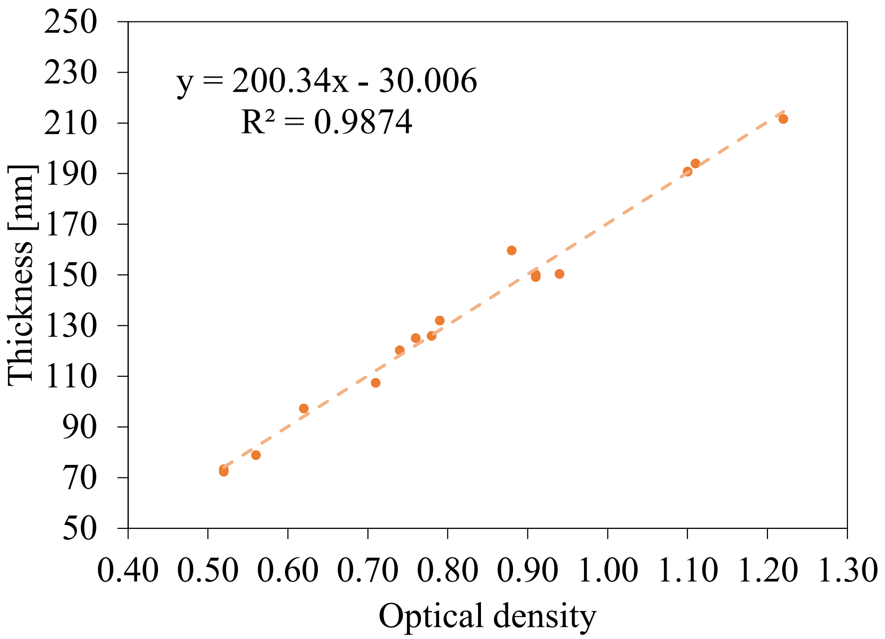

Figure S 9. Measured thickness of uAJP donor films printed under the same BBD conditions over plain glass versus the OD donor peak intensity.

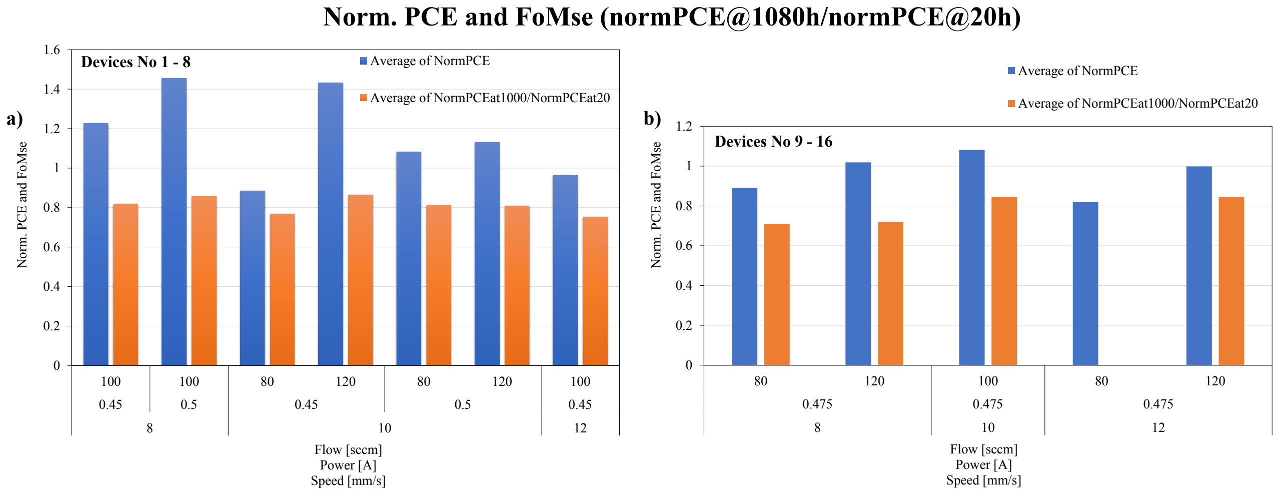

Figure S 10. Normalized PCE and FoMse under one sun illumination before and after 1080h degradation on a) number 1-8 and b) number 9-16 inverted OPV devices with ultrasonic aerosol jet printed donor material

Most devices exhibited an initial increase in PCE within the first 20 hours. To more accurately quantify long-term stability, we defined a Figure of Merit for stability enhancement (FoMse),

calculated as the ratio of normalized PCE at 1080 h to that at 20 h. Although light soaking of the ZnO electron transport layer (ETL) can cause performance enhancement, this effect typically occurs within seconds or minutes, not over hours. Additionally, the absence of s-shaped J–V curves at 0 h suggests that ETL light soaking is unlikely the cause. Therefore, the observed PCE improvement may be due to morphological changes in the active layer during aging, though further in-depth studies are required to confirm this hypothesis. Notably, most devices with FoMse values below 0.8 were fabricated using a flow rate of 80 sccm, indicating that insufficient donor deposition may correlate with reduced stability. The device processed at 80 sccm, 0.475 A, and 12 mm/s could not be measured, likely due to a chamber issue with the pin connections at that specific position. In the degradation setup, it exhibited no measurable PCE after long-term testing. However, when measured under AM1.5G in LineOne, it still showed some efficiency.

```
                            OLS Regression Results
==============================================================================
Dep. Variable:                  Ratio   R-squared:                       0.996
Model:                            OLS   Adj. R-squared:                  0.990
Method:                 Least Squares   F-statistic:                     165.6
Date:                Mon, 13 Jan 2025   Prob (F-statistic):           1.71e-06
Time:                        10:22:33   Log-Likelihood:                 42.036
No. Observations:                  16   AIC:                            -64.07
Df Residuals:                       6   BIC:                            -56.35
Df Model:                           9
Covariance Type:            nonrobust
=====================================================================================
                        coef    std err          t      P>|t|      [0.025      0.975]
-------------------------------------------------------------------------------------
Intercept            27.7307      4.149      6.684      0.001      17.580      37.882
Power                14.4343      2.211      6.529      0.001       9.025      19.844
Speed                -4.5513      0.939     -4.846      0.003      -6.849      -2.253
Power:Speed          10.3179      1.972      5.233      0.002       5.493      15.142
Flow                 -0.6098      0.094     -6.491      0.001      -0.840      -0.380
Power:Flow            1.1227      0.197      5.693      0.001       0.640       1.605
Speed:Flow            0.0521      0.009      5.506      0.002       0.029       0.075
Power:Speed:Flow     -0.1063      0.020     -5.338      0.002      -0.155      -0.058
I(Power ** 2)      -136.2263     22.847     -5.963      0.001    -192.131     -80.322
I(Speed ** 2)        -0.0234      0.004     -6.542      0.001      -0.032      -0.015
I(Flow ** 2)          0.0002   3.57e-05      6.152      0.001       0.000       0.000
...
Notes:
[1] Standard Errors assume that the covariance matrix of the errors is correctly specified.
[2] The smallest eigenvalue is 5.68e-26. This might indicate that there are
strong multicollinearity problems or that the design matrix is singular.
Output is truncated. View as a scrollable element or open in a text editor. Adjust cell output settings...
c:\Users\ArangoV\AppData\Local\miniconda3\envs\amandadata\Lib\site-packages\scipy\stats\_axis
  return hypotest_fun_in(*args, **kwds)
```

Figure S 11. Statistical analysis of Ratio as the control variable on the BBD

```
                            OLS Regression Results
==============================================================================
Dep. Variable:                    PCE   R-squared:                       0.973
Model:                            OLS   Adj. R-squared:                  0.933
Method:                 Least Squares   F-statistic:                     24.26
Date:                Mon, 03 Feb 2025   Prob (F-statistic):           0.000478
Time:                        13:44:01   Log-Likelihood:                -7.4564
No. Observations:                  16   AIC:                             34.91
Df Residuals:                       6   BIC:                             42.64
Df Model:                           9
Covariance Type:            nonrobust
====================================================================================
                       coef    std err          t      P>|t|      [0.025      0.975]
------------------------------------------------------------------------------------
Intercept          420.5017     91.474      4.597      0.004     196.674     644.330
Power              227.4496     48.744      4.666      0.003     108.177     346.722
Speed             -106.0708     20.709     -5.122      0.002    -156.743     -55.398
Power:Speed        223.7836     43.474      5.148      0.002     117.407     330.160
Flow               -10.7813      2.071     -5.205      0.002     -15.850      -5.713
Power:Flow          24.0147      4.348      5.523      0.001      13.375      34.655
Speed:Flow           1.2656      0.209      6.063      0.001       0.755       1.776
Power:Speed:Flow    -2.5675      0.439     -5.845      0.001      -3.642      -1.493
I(Power ** 2)    -2448.8546    503.768     -4.861      0.003   -3681.530   -1216.179
I(Speed ** 2)       -0.2602      0.079     -3.305      0.016      -0.453      -0.068
I(Flow ** 2)        -0.0050      0.001     -6.381      0.001      -0.007      -0.003
...
Notes:
[1] Standard Errors assume that the covariance matrix of the errors is correctly specified.
[2] The smallest eigenvalue is 5.68e-26. This might indicate that there are
strong multicollinearity problems or that the design matrix is singular.
Output is truncated. View as a scrollable element or open in a text editor. Adjust cell output settings...
c:\Users\ArangoV\AppData\Local\miniconda3\envs\amandadata\Lib\site-packages\scipy\stats\_axis
  return hypotest_fun_in(*args, **kwds)
```

Figure S 12. Statistical analysis of PCE as the control variable on the BBD

```
                            OLS Regression Results
==============================================================================
Dep. Variable:                    Jsc   R-squared:                       0.989
Model:                            OLS   Adj. R-squared:                  0.972
Method:                 Least Squares   F-statistic:                     58.65
Date:                Mon, 03 Feb 2025   Prob (F-statistic):           3.70e-05
Time:                        15:30:24   Log-Likelihood:                -10.510
No. Observations:                  16   AIC:                             41.02
Df Residuals:                       6   BIC:                             48.75
Df Model:                           9
Covariance Type:            nonrobust
====================================================================================
                       coef    std err          t      P>|t|      [0.025      0.975]
------------------------------------------------------------------------------------
Intercept          754.8488    110.708      6.818      0.000     483.955    1025.743
Power              409.7155     58.994      6.945      0.000     265.363     554.068
Speed             -194.0192     25.063     -7.741      0.000    -255.347    -132.691
Power:Speed        413.8257     52.615      7.865      0.000     285.080     542.571
Flow               -20.2039      2.507     -8.059      0.000     -26.338     -14.070
Power:Flow          46.1922      5.263      8.777      0.000      33.315      59.069
Speed:Flow           2.3347      0.253      9.241      0.000       1.716       2.953
Power:Speed:Flow    -4.8298      0.532     -9.085      0.000      -6.131      -3.529
I(Power ** 2)    -4580.1968    609.698     -7.512      0.000   -6072.074   -3088.320
I(Speed ** 2)       -0.3512      0.095     -3.686      0.010      -0.584      -0.118
I(Flow ** 2)        -0.0097      0.001    -10.160      0.000      -0.012      -0.007
...
Notes:
[1] Standard Errors assume that the covariance matrix of the errors is correctly specified.
[2] The smallest eigenvalue is 5.68e-26. This might indicate that there are
strong multicollinearity problems or that the design matrix is singular.
Output is truncated. View as a scrollable element or open in a text editor. Adjust cell output settings...
c:\Users\ArangoV\AppData\Local\miniconda3\envs\amandadata\Lib\site-packages\scipy\stats\_axis
  return hypotest_fun_in(*args, **kwds)
```

Figure S 13. Statistical analysis of Jsc as the control variable on the BBD

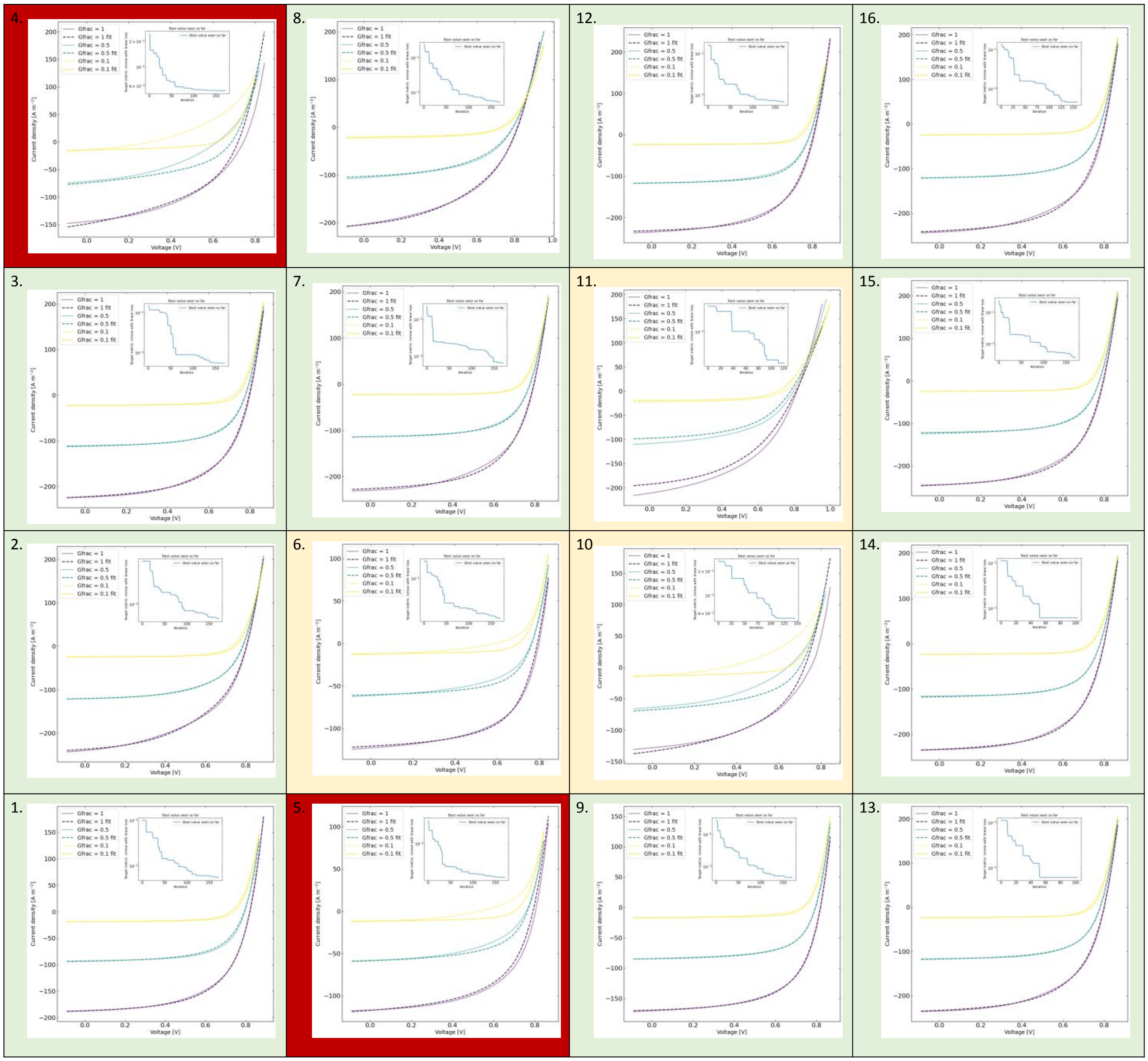

Figure S 14. JV curves real (continuous lines) and fitted with DD (dashed lines) of the best cell per OPV device. The device number on the BBD DoE is on the corner up left.

The fitting parameters were electron mobility (μ□) and hole mobility (μ□) in $m^2V^{-1}s^{-1}$, bulk defect density (N□_bulk) in m^{-3}, Langevin recombination efficiency (preLangevin) in m^3s^{-1}, series resistance (R_series) in $\Omega \cdot m^2$, shunt resistance (R_shunt) in $\Omega \cdot m^2$, effective charge generation rate (G_ehp) in $m^{-3}s^{-1}$, and the work function of the hole transport layer (W_R) in eV.

Table S 1. Fitted Values with Drift Diffusion

Device	mu_n	mu_p	N_t_bulk	preLangevin	R_series	R_shunt	G_ehp	W_R
1	1.08E-07	5.74E-07	1.93E+21	0.0040948218	3.74E-05	52.55431199	6.90E+27	5.378680151
2	7.86E-07	5.90E-08	3.77E+18	0.0502169500	3.44E-05	88.11047893	7.06E+27	5.468656744
3	5.01E-08	7.99E-07	6.17E+18	0.1077155349	7.48E-05	24.19279369	1.11E+28	5.414408621
4	4.45E-07	3.48E-07	2.33E+22	0.0635402850	1.90E-06	47.97551384	7.75E+27	5.320507275
5	1.31E-07	3.73E-08	5.64E+21	0.0436035600	8.20E-07	1286610.354	8.46E+27	5.314229901
6	4.93E-08	1.63E-07	4.91E+21	0.0677854305	0.000112968	35.54174086	8.27E+27	5.374760057
7	4.95E-07	9.49E-08	2.45E+20	0.1189206019	4.00E-06	10.34389979	8.38E+27	5.353866831
8	3.37E-07	4.26E-08	1.70E+21	0.0182282609	6.76E-05	25.33139598	6.65E+27	5.346891525
9	4.14E-07	3.52E-08	2.32E+21	0.0357989934	4.90E-05	37.93606093	1.03E+28	5.474222106
10	9.57E-07	3.13E-08	2.14E+22	0.8930634751	9.88E-05	12.33856047	1.04E+28	5.368425854
11	1.43E-07	4.38E-07	2.09E+21	0.0012819607	0.000600751	81.90016375	5.63E+27	5.47659512
12	5.15E-07	9.51E-08	9.19E+16	0.0741443584	1.73E-05	19.87777957	7.95E+27	5.322707837
13	5.33E-07	6.74E-08	3.28E+20	0.1110610135	2.78E-05	8.556823792	1.02E+28	5.32302891
14	1.13E-07	4.57E-07	6.64E+20	0.1395874335	3.40E-05	5.504693904	1.06E+28	5.373005672
15	5.36E-07	8.84E-08	3.82E+20	0.1052461575	3.20E-05	56.1268667	9.50E+27	5.309253531
16	9.98E-07	5.53E-08	4.75E+20	0.0646919081	7.58E-05	69.81289171	1.02E+28	5.309915039

Abs data PIDs = [41846, 41847, 41848, 41849, 41850, 41851, 41852, 41853, 41870, 41871, 41872, 41873, 41874, 41875, 41876, 41877]; IV data PIDs = [43159, 43160, 43161, 43162, 43163, 43164, 43165, 43166, 43167, 43168, 43169, 43170, 43171, 43172, 43173, 43174] 39502.

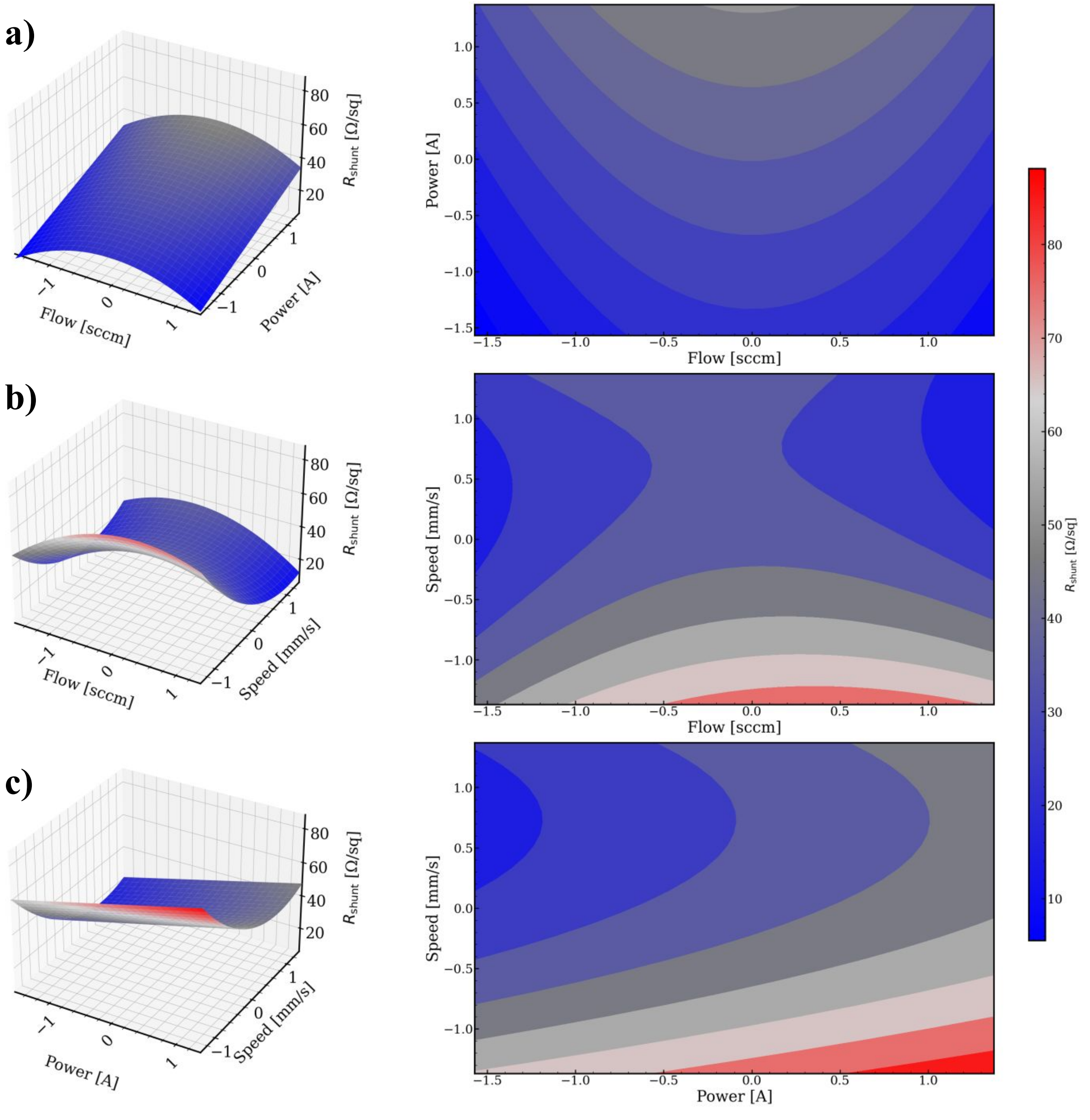

Figure S 15. Response surface method with Box-Behnken design of experiments using R_{shunt} as the control variable. Left side: Response surface plots, and right side: Contour plots. Screening two factors of a) flow and power, b) flow and speed and c) power and speed

```
Final predictors used: ['Intercept', 'Power', 'Speed', 'I(Speed * Flow)', 'I(Speed ** 2)', 'I(Flow ** 2)']
                            OLS Regression Results
==============================================================================
Dep. Variable:                R_shunt   R-squared:                       0.615
Model:                            OLS   Adj. R-squared:                  0.401
Method:                 Least Squares   F-statistic:                     2.876
Date:                Do, 08 Mai 2025   Prob (F-statistic):             0.0801
Time:                        19:37:15   Log-Likelihood:                -62.980
No. Observations:                  15   AIC:                             138.0
Df Residuals:                       9   BIC:                             142.2
Df Model:                           5
Covariance Type:            nonrobust
===================================================================================
                      coef    std err          t      P>|t|      [0.025      0.975]
-----------------------------------------------------------------------------------
Intercept          36.1492      9.053      3.993      0.003      15.669      56.629
Power               9.1144      5.458      1.670      0.129      -3.233      21.461
Speed             -14.6948      5.395     -2.724      0.023     -26.900      -2.490
I(Speed * Flow)    -4.5213      5.165     -0.875      0.404     -16.204       7.162
I(Speed ** 2)      10.0698      5.807      1.734      0.117      -3.066      23.206
I(Flow ** 2)       -7.8121      5.063     -1.543      0.157     -19.265       3.641
==============================================================================
Omnibus:                        0.451   Durbin-Watson:                   1.292
Prob(Omnibus):                  0.798   Jarque-Bera (JB):                0.025
Skew:                           0.098   Prob(JB):                        0.987
...
==============================================================================

Notes:
[1] Standard Errors assume that the covariance matrix of the errors is correctly specified.
Output is truncated. View as a scrollable element or open in a text editor. Adjust cell output settings...
c:\Users\ArangoV\AppData\Local\miniconda3\envs\amandadata\Lib\site-packages\scipy\stats\_axis_nan_policy.py:
  return hypotest_fun_in(*args, **kwds)
```

Figure S 16. Statistical analysis of R_{shunt} as the control variable on the BBD

For the R_{shunt} plotting and analysis, we excluded the results from the device 5, due to unsatisfactory fits, which might be related to the combined low conditions of flow and power at middle speed while AJP deposition. Additionally, the devices 4, 6, 10, and 11, which showed low efficiencies, also showed poor fits using one-dimensional drift-diffusion model.
